# Genome-wide admixture and association study of subclinical atherosclerosis in the Women’s Interagency HIV Study (WIHS)

**DOI:** 10.1371/journal.pone.0188725

**Published:** 2017-12-04

**Authors:** Aditi Shendre, Howard W. Wiener, Marguerite R. Irvin, Bradley E. Aouizerat, Edgar T. Overton, Jason Lazar, Chenglong Liu, Howard N. Hodis, Nita A. Limdi, Kathleen M. Weber, Stephen J. Gange, Degui Zhi, Michelle A. Floris-Moore, Ighovwerha Ofotokun, Qibin Qi, David B. Hanna, Robert C. Kaplan, Sadeep Shrestha

**Affiliations:** 1 Department of Epidemiology, University of Alabama at Birmingham, Birmingham, Alabama, United States of America; 2 Bluestone Center for Clinical Research, New York University, New York, New York, United States of America; 3 Department of Oral and Maxillofacial Surgery, New York University, New York, New York, United States of America; 4 Division of Infectious Diseases, Department of Medicine, University of Alabama at Birmingham, Birmingham, Alabama, United States of America; 5 Department of Medicine, State University of New York, Downstate Medical Center, Brooklyn, New York, United States of America; 6 Department of Medicine, Georgetown University Medical Center, Washington, DC, United States of America; 7 Atherosclerosis Research Unit, University of Southern California, Los Angeles, California, United States of America; 8 Department of Neurology, University of Alabama at Birmingham, Birmingham, Alabama, United States of America; 9 Cook County Health and Hospital System/Hektoen Institute of Medicine, Chicago, Illnois, United States of America; 10 Department of Epidemiology, Johns Hopkins Bloomberg School of Public Health, Baltimore, Maryland, United States of America; 11 Department of Biostatistics, University of Alabama at Birmingham, Birmingham, Alabama, United States of America; 12 Division of Infectious Diseases, Department of Medicine, University of North Carolina School of Medicine, Chapel Hill, North Carolina, United States of America; 13 Department of Medicine/Infectious Diseases, Emory University, and Grady Healthcare System, Atlanta, Georgia, United States of America; 14 Department of Epidemiology and Population Health, Albert Einstein College of Medicine, Bronx, New York, United States of America; Centro Cardiologico Monzino, ITALY

## Abstract

Cardiovascular disease (CVD) is a major comorbidity among HIV-infected individuals. Common carotid artery intima-media thickness (cCIMT) is a valid and reliable subclinical measure of atherosclerosis and is known to predict CVD. We performed genome-wide association (GWA) and admixture analysis among 682 HIV-positive and 288 HIV-negative Black, non-Hispanic women from the Women’s Interagency HIV study (WIHS) cohort using a combined and stratified analysis approach. We found some suggestive associations but none of the SNPs reached genome-wide statistical significance in our GWAS analysis. The top GWAS SNPs were rs2280828 in the region intergenic to mediator complex subunit 30 and exostosin glycosyltransferase 1 (*MED30* | *EXT1*) among all women, rs2907092 in the catenin delta 2 (*CTNND2*) gene among HIV-positive women, and rs7529733 in the region intergenic to family with sequence similarity 5, member C and regulator of G-protein signaling 18 (*FAM5C* | *RGS18*) genes among HIV-negative women. The most significant local European ancestry associations were in the region intergenic to the zinc finger and SCAN domain containing 5D gene and NADH: ubiquinone oxidoreductase complex assembly factor 1 (*ZSCAN5D* | *NDUF1*) pseudogene on chromosome 19 among all women, in the region intergenic to vomeronasal 1 receptor 6 pseudogene and zinc finger protein 845 (*VN1R6P* | *ZNF845*) gene on chromosome 19 among HIV-positive women, and in the region intergenic to the SEC23-interacting protein and phosphatidic acid phosphatase type 2 domain containing 1A (*SEC23IP* | *PPAPDC1A*) genes located on chromosome 10 among HIV-negative women. A number of previously identified SNP associations with cCIMT were also observed and included rs2572204 in the ryanodine receptor 3 (*RYR3*) and an admixture region in the secretion-regulating guanine nucleotide exchange factor (*SERGEF*) gene. We report several SNPs and gene regions in the GWAS and admixture analysis, some of which are common across HIV-positive and HIV-negative women as demonstrated using meta-analysis, and also across the two analytic approaches (i.e., GWA and admixture). These findings suggest that local European ancestry plays an important role in genetic associations of cCIMT among black women from WIHS along with other environmental factors that are related to CVD and may also be triggered by HIV. These findings warrant confirmation in independent samples.

## Introduction

Early diagnosis and treatment of human immunodeficiency virus (HIV) has increased the life expectancy of infected individuals [1], and shifted the focus to the management of other comorbidities such as cardiovascular disease (CVD). CVD risk is higher among HIV-positive individuals compared to HIV-negative individuals [2–4]. HIV infection as well as certain antiretroviral therapies (ART) were shown to independently increase the risk of CVD [2]. Moreover, disparities in CVD risk by race and sex prevail, specifically, among HIV-positive individuals [5, 6]. Higher rates of acute myocardial infarction were observed among Blacks as compared to Whites, and among women as compared to men [3–5]. Similarly, ischemic stroke rates were reported to be higher among HIV-positive women as compared to HIV-positive men [7].

Efforts to prevent CVD among HIV-positive individuals have led to the evaluation of subclinical atherosclerotic measures such as carotid artery intima-media thickness (CIMT). CIMT can predict future CVD events among HIV-negative individuals [8]. CIMT is a non-invasive measure obtained using B-mode ultrasound, with the common carotid artery measurements considered the most reliable and reproducible of all CIMT segments [9, 10]. Common CIMT (cCIMT) measurements were reported to be thicker among HIV-positive as compared to matched HIV-negative individuals, and also independently associated with HIV infection and treatment [11, 12]. Racial and sex differences have shown greater cCIMT thickness among Blacks as compared to Whites, and among women as compared to men beyond traditional CVD risk factors [13–15].

The role of genetics as a risk factor for subclinical atherosclerosis, specifically in genome-wide association studies (GWAS) of cCIMT, has been explored among the HIV-negative [16] and non-Black populations [16–18]. A recent study on subclinical atherosclerosis that conducted a exome-wide association analysis, reported two SNPs–rs7412 in the apolipoprotein E (*APOE*) gene and rs143873045 in the KN Motif and Ankyrin Repeat Domains 2 (*KANK2*) gene that were found significantly associated with cCIMT among HIV-negative Blacks [19]. Among HIV-positive individuals, the only GWAS in relation to cCIMT has been conducted by our group, among White men from the Fat Redistribution and Metabolic Change in HIV Infection (FRAM) Study [20]. We observed two single nucleotide polymorphisms (SNPs) in tight linkage disequilibrium (LD), rs2229116 and rs7177922, in the ryanodine receptor 3 (*RYR3*) gene on chromosome 15 to be associated with cCIMT. The association of rs2229116 was replicated in another group of HIV-positive White men from the Multicenter AIDS Cohort Study (MACS) [21]. Given the consistent association with cCIMT in HIV-positive White men, we further evaluated the association of SNPs within the *RYR3* gene among a racially diverse group of HIV-positive women from the Women’s Interagency HIV Study (WIHS) [22]. The *RYR3* gene association with cCIMT was observed among different SNPs than those reported earlier, rs2572204 being most significant among Black HIV-positive women. Haplotype blocks observed among the Black HIV-positive women compared to the other two racial/ethnic groups were smaller and indicated that SNP associations are possibly different based on LD, warranting further evaluation specifically in the context of cCIMT related admixture.

Admixture mapping enables the identification of genetic variations differentially distributed in ancestral populations associated with a disease–the premise being that associated variants are observed on chromosomal segments from ancestral populations with higher prevalence of the disease/trait [23]. Thus, this approach takes advantage of the extended LD present in the recently admixed population to discover these variations. Admixture mapping has been considered a feasible and more powerful tool in contrast to GWAS among individuals of mixed continental ancestry [24], and advances in statistical techniques and their applications in a genome-wide set of markers has greatly increased the ability to detect disease loci. We recently performed genome-wide admixture mapping in relation to cCIMT among HIV-negative Black individuals from two large prospective cohorts and found a region in the secretion regulating guanine nucleotide exchange factor (*SERGEF*) gene that reached genome-wide significance [25]. Likewise, we have shown various genomic regions associated with clinical events in African Americans through admixture mapping [26].

Currently, methods based on principal components are commonly used to control for population stratification in genetic association studies [27]. Recent studies have focused on admixture mapping and related techniques to incorporate ancestry information in association tests among admixed populations [28–32]. However, there is a lack of consensus in approaches and whether accounting for local and global ancestries alone or together would sufficiently control for both type I and type II error rates [31, 32]. In this study, we performed genome-wide admixture and association analysis to determine single SNP and local European ancestry associations with cCIMT among Black women from the WIHS cohort.

## Results

The HIV and CVD related characteristics of the 970 Black women included in the current study are presented in **Table 1**. In this study, 70% of the women were HIV-positive, and had similar cCIMT values as well as percent global European ancestry as compared to HIV-negative women. CVD risk factors including hypertension, diabetes, and current smoking were similar across the two groups. HIV-positive women were older, had lower CD4 cell count and HDL cholesterol levels, and were more likely to take medications for hypertension compared to HIV-negative women.

**Table 1:** Characteristics of all women from the Women’s Interagency HIV Study (WIHS) cohort and by HIV status.

Characteristics	All (N = 970)	HIV positive (N = 682)	HIV negative (N = 288)
cCIMT (mm), mean (SD)	0.74 (0.12)	0.75 (0.12)	0.74 (0.12)
Percent global European ancestry, mean (SD)	15.7 (8.5)	15.7 (8.4)	15.7 (8.6)
Age (years), mean (SD)	41.1 (9.3)	41.9 (8.8) **	39.1 (10.0)
CD4 cell count (cells/mm^3^), median (IQR)	577 (559)	436 (393) **	1029 (516)
Anti-retroviral therapy, n (%)	-	633 (92.3)	-
Hypertension, n (%)	343 (35.4)	250 (36.7)	93 (32.3)
Diabetes, n (%)	135 (13.9)	93 (13.6)	42 (14.6)
Current smoking, n (%)	504 (51.9)	352 (51.6)	152 (52.8)
HDL cholesterol (mg/dL), mean (SD)	51.3 (18.1)	49.2 (18.2) **	56.1 (16.7)
LDL cholesterol (mg/dL), mean (SD)	100.1 (33.7)	98.9 (33.1)	102.9 (35.0)

HIV: human immunodeficiency virus, SD: standard deviation, IQR: inter-quartile range, HDL: high density lipoprotein, LDL: low density lipoprotein

*P<0.01

**P<0.001

### Genome-wide association results

**Fig 1:**
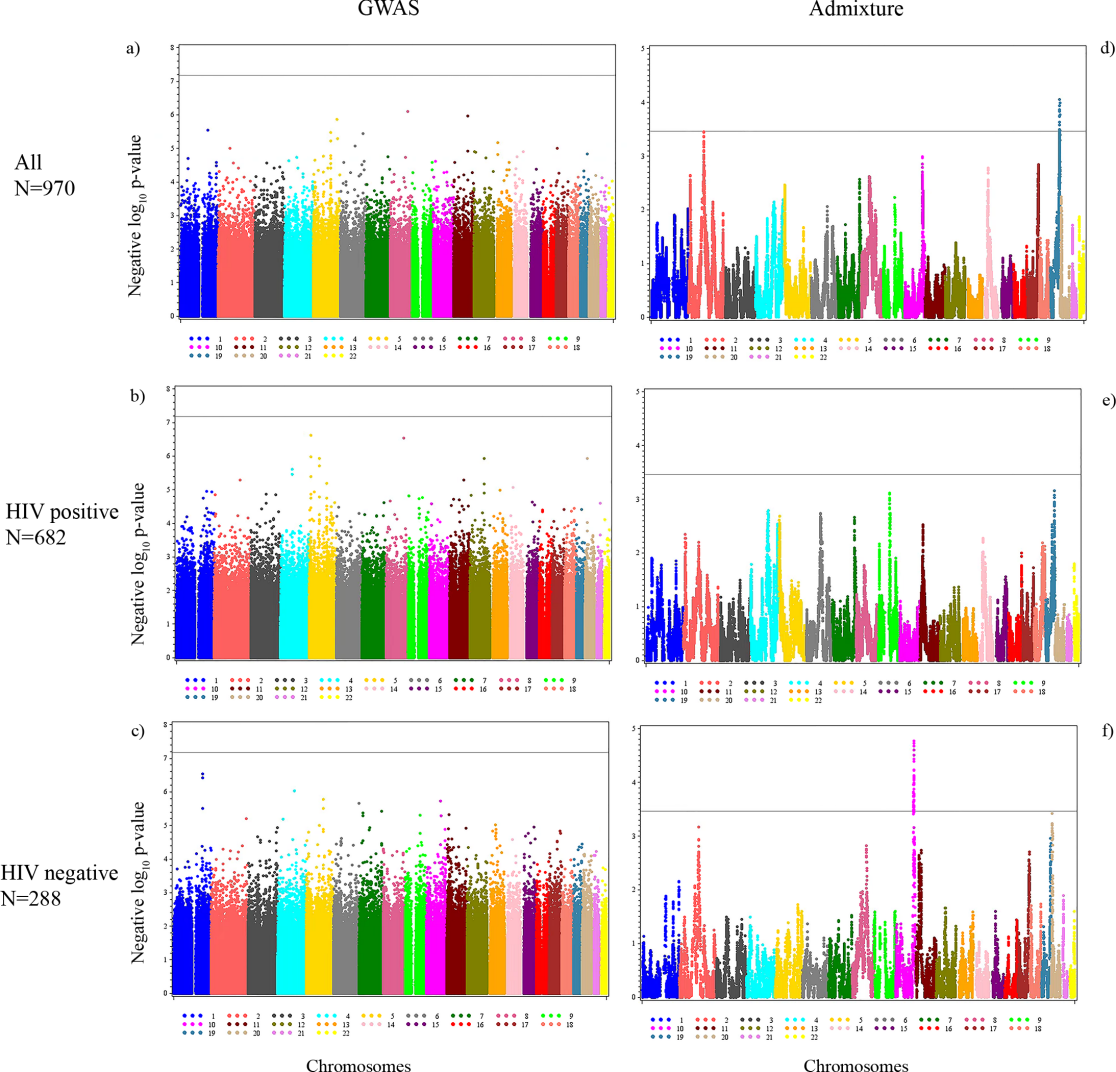
Manhattan plots for the genome-wide association (threshold p-value = 6.50×10^−8^) and admixture analyses (threshold p-value 3.42×10^−4^). a) All women (GWAS), b) HIV-positive women (GWAS), c) HIV-negative women (GWAS), d) All women (admixture), e) HIV-positive women (admixture), and f) HIV-negative women (admixture). Negative log_10_ p-values are plotted against each SNP’s respective position on each chromosome.

**Fig 2:**
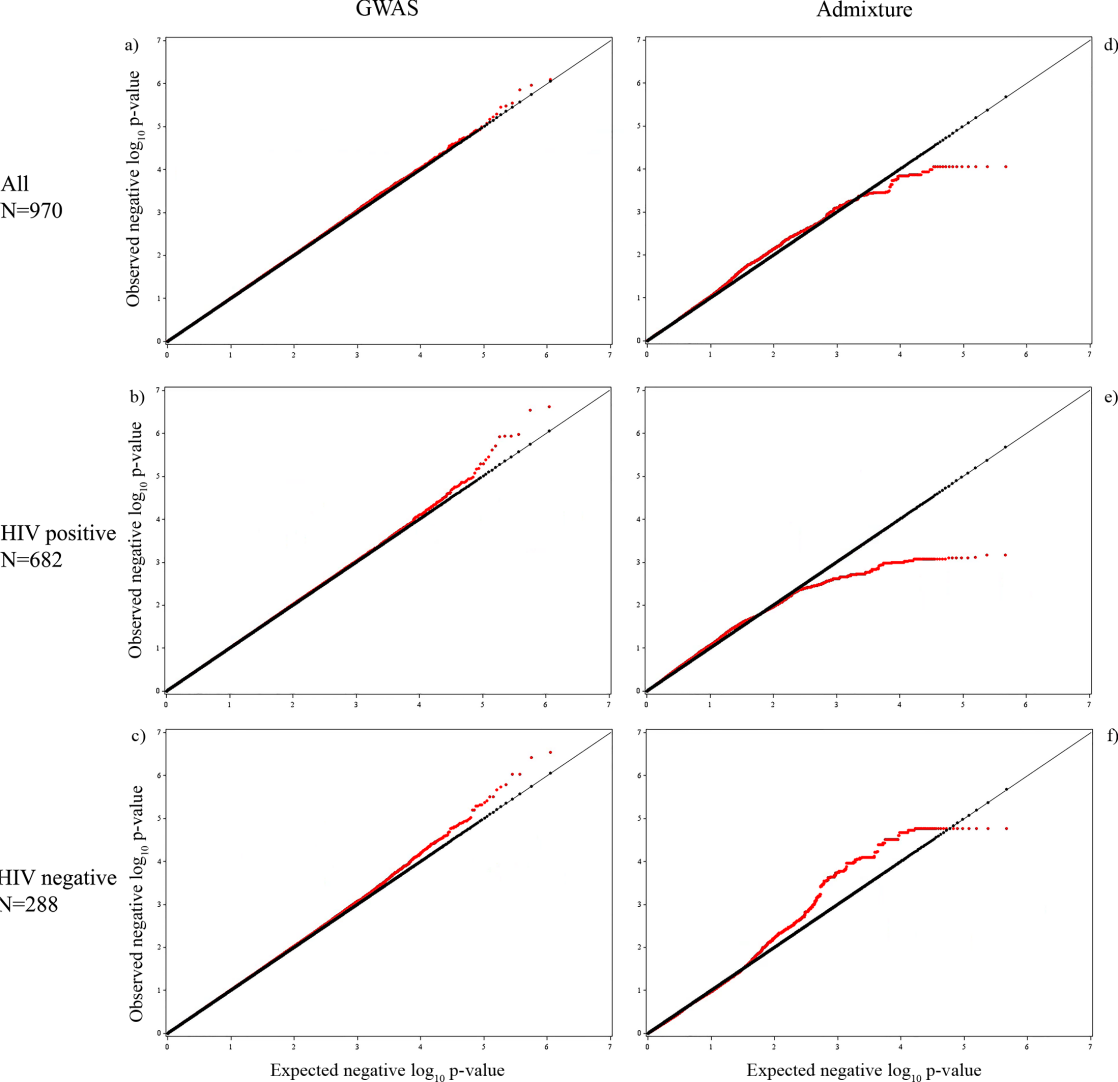
QQ plots for the genome-wide association analyses and admixture analyses. a) All women (GWAS, λ = 1.005), b) HIV-positive women (GWAS, λ = 1.009), c) HIV-negative women (GWAS, λ = 1.013), d) All women (admixture, λ = 1.001), e) HIV-positive women (admixture, λ = 1.103), and f) HIV-negative women (admixture, λ = 1.000). Observed negative log10 p-values are plotted against the expected negative log10 p-values.

The genome-wide association results for cCIMT are presented for all women and after further stratification by HIV status, as shown in the Manhattan plots (**Fig 1A–1C**). None of the SNPs reached the genome-wide significance level (**S1 Table**), but we present the top 5 SNP-cCIMT associations for all three groups (**Table 2**) and the regionals plots for the top SNPs in each group **(S1A–S1C Fig)**. The lambda values for each of the GWAS models were– 1.005 for the combined model, 1.009 for the HIV-positive model, and 1.013 for the HIV-negative model (**Fig 2A–2C**). We also present the admixture analyses results for gene regions that include these significant GWAS SNPs to determine if local European ancestry at these regions is associated with cCIMT in **Table 2**. The top hit among all women was rs2280828 located in the intergenic region of mediator complex subunit 30 and exostosin glycosyltransferase 1 (*MED30* | *EXT1*) genes on chromosome 8 (β = -0.0216, p = 8.01x10^-7^). The same SNP was also significantly associated with cCIMT among HIV-positive women (β = -0.0271, p = 2.89x10^-7^). The most significant SNP associated with cCIMT among HIV-positive women was rs2907092 in the catenin delta 2 (*CTNND2*) gene on chromosome 5 (β = 0.0250, p = 2.37x10^-7^). Local European ancestry at the corresponding gene region of this SNP was also associated with cCIMT (β = 0.0089, p = 0.04). Local European ancestry was also associated with cCIMT at the gene region that includes the GWAS identified rs4761669 intergenic to the transmembrane and coiled-coil domain family 3 gene and the NADH: ubiquinone oxidoreductase subunit A12 (*TMCC3* | *NDUFA12*) genes on chromosome 12 (β = -0.0084, p = 0.04). Among HIV-negative women, rs7529733 intergenic to family with sequence similarity 5, member C and regulator of G-protein signaling 18 genes (*FAM5C* | *RGS18*) on chromosome 1 was the top SNP associated with cCIMT (β = 0.0431, p = 2.92x10^-7^).

**Table 2:** Genome-wide association (GWA) results for common carotid artery intima media thickness and the corresponding admixture (LEA) regions for the top 5 hits among participants from the Women’s Interagency HIV Study (WIHS) cohort.

	GWA							Comparison in Admixture				
Chromosome	Position	SNP	MAF- CEU/YRI	Gene	Location	Beta	*P*	Gene region	First SNP	Last SNP	Beta	*P*
**Combined**												
8	118762436	rs2280828	C: 0.009/0.08	*MED30 | EXT1*	Intergenic	-0.0216	**8.0E-07**	118.554–118.808	rs16889862	rs4876390	-0.0038	0.30
11	94908658	rs11021065	T: 0.27/0.18	*SESN3*	Intron	0.0149	1.1E-06	94.908–94.922	rs11021065	rs629508	0.0030	0.39
5	159844275	rs2910195	C: 0.09/0.06	*SLU7*	Intron	0.0218	1.4E-06	159.84–159.846	rs2961949	rs17057781	-0.0017	0.64
1	182404684	rs557514	A: 0.38/0.33	*C1orf120 | RGSL1*	Intergenic	-0.0122	2.9E-06	182.385–182.41	rs6687401	rs6677117	-0.0029	0.39
5	117982046	rs2914908	G: 0.50/0.28	*LOC100287135 | DTWD2*	Intergenic	-0.0117	3.3E-06	116.369–118.167	rs17140968	rs2081957	-0.0039	0.26
**HIV-positive**												
5	11047681	rs2907092	G: 0.27/0.13	*CTNND2*	Intron	0.0250	**2.4E-07**	10.973–11.903	rs1566622	rs1458473	0.0089	**0.04**
8	118762436	rs2280828	C: 0.009/0.08	*MED30 | EXT1*	Intergenic	-0.0271	**2.9E-07**	118.554–118.808	rs16889862	rs4876390	-0.0023	0.60
12	95187622	rs4761669	G: 0.27/0.04	*TMCC3 | NDUFA12*	Intergenic	-0.0273	1.2E-06	95.046–95.363	rs2651982	rs10859809	-0.0084	**0.04**
5	66543279	rs73112098	G: 0.005/0.13	*CD180 | PIK3R1*	Intergenic	0.0222	1.2E-06	66.492–67.517	rs3733911	rs831227	-0.0018	0.66
20	14911683	rs11087114	A: 0.14/0.06	*MACROD2*	Intron	0.0273	1.2E-06	13.977–16.033	rs6110117	rs1238278	0.0020	0.64
**HIV-negative**												
1	190811488	rs7529733	A: 0.006/0.05	*FAM5C | RGS18*	Intergenic	0.0431	**2.9E-07**	190.467–192.123	rs703931	rs7526348	0.0029	0.67
4	110081021	rs11931270	A:0.008/0.04	*COL25A1*	Intron	0.0444	**9.3E-07**	109.75–110.223	rs1452689	rs1859143	-0.0088	0.18
5	111526308	rs17134212	T: 0.23/0.07	*EPB41L4A*	Intron	-0.0276	1.7E-06	111.499–111.75	rs12517400	rs17134502	0.0023	0.71
10	96110492	rs76426033	A: -/-	*NOC3L*	Intron	0.0359	1.8E-06	96.114–96.122	rs12572897	rs11187904	-0.0052	0.39
6	169585451	rs114413935	T: 0.00/0.18	*SMOC2 | THBS2*	Intergenic	0.0296	2.2E-06	169.075–169.607	rs1885634	rs7764005	0.0072	0.26

SNP: single nucleotide polymorphisms, LEA: local European ancestry

### Local ancestry association results

The local European ancestry (LEA) association with cCIMT is presented in **Table 3** for the top 5 gene regions for all women and then separately for HIV-positive and HIV-negative women, and the corresponding Manhattan plots are presented in **Fig 1D**–**1F.** The regionals plots for the top regions are presented in **S1D–S1F Fig** (**all regions in S2 Table**). The lambda values for each of the admixture models were– 1.001 for the combined model, 1.103 for the HIV-positive model, and 1.000 for the HIV-negative model (**Fig 2D–2F**). We also present the top GWAS hit located within the top ancestry gene regions if it was significant at α of 0.05 (**Table 3)**. LEA at 17 gene regions reached genome-wide significance in relation to cCIMT among all women. These regions were largely located on chromosome 19 over a 3.07 Mb region but also included one gene region on chromosome 6 spanning 106.38 kb. There were no gene regions that reached genome-wide significance among HIV-positive women. Among HIV-negative women, a total of 48 LEA gene regions achieved genome-wide significance and were largely located on chromosome 10 over a span of 4.23 Mb. A single gene region each on chromosomes 2, 4, and 9 also achieved genome-wide significance among HIV-negative women. The most significant local European ancestry association among all women was observed in the region intergenic to the zinc finger and SCAN domain containing 5D gene and NADH: ubiquinone oxidoreductase complex assembly factor 1 pseudogene (*ZSCAN5D | NDUF1*) on chromosome 19 (β = 0.0134, p = 8.87x10^-5^). The region on chromosome 6 in the leucine rich repeat containing 1 gene (*LRRC1*) achieved genome-wide significance among all women but was also observed among the top 5 regions in HIV-positive women (β = 0.0138, p = 8.42x10^-4^). The region intergenic to the vomeronasal 1 receptor 6 pseudogene and zinc finger protein 845 gene (*VN1R6P | ZNF845*) on chromosome 19 was most significant among HIV-positive women (β = 0.0141, p = 6.86x10^-4^). Among HIV-negative women, the top association was observed in the region intergenic to the SEC23 interacting protein gene and phosphatidic acid phosphatase type 2 domain containing 1A gene located on chromosome 10 (*SEC23IP | PPAPDC1A*) (β = -0.0290, p = 1.71x10^-5^).

**Table 3:** Genome-wide admixture (LEA) and the corresponding genome wide association (GWA) results for the top 5 hits among participants from the Women’s Interagency HIV Study (WIHS) cohort.

Admixture							Comparison in GWA					
Chromosome	Gene region	First SNP	Last SNP	Gene	Beta	*P*	Position	SNP	MAF- CEU/YRI	Location	Beta	*P*
**Combined**												
19	56.761–56.832	rs10420275	rs11881864	*ZSCAN5D | NDUF1*	0.0134	8.9E-05	56777293	rs12978423	A:0.75/0.42	Intergenic	0.0112	0.008
19	56.689–56.696	rs16987032	rs7258532	*GALP*	0.0135	8.9E-05	56693265	rs73934760	C: 0.00/0.03	Intron	-0.0130	0.005*
19	56.698–56.698	rs4488567	rs11084425	*GALP | ZSCAN5B*	0.0135	8.9E-05	56698547	rs11084425	G: 0.23/0.04	Intergenic	0.0145	0.005*
19	56.657–56.671	rs274179	rs274154	*ZNF444*	0.0135	8.9E-05	‒	‒	‒	‒	‒	‒
19	56.573–56.595	rs430931	rs11672395	*NLRP5*	0.0132	1.4E-04	‒	‒	‒	‒	‒	‒
**HIV-positive**												
19	53.82–53.833	rs8104591	rs11672485	*VN1R6P | ZNF845*	0.0141	6.9E-04	‒	‒	‒	‒	‒	‒
9	77.305–77.332	rs1410227	rs1536613	*RORB | TRPM6*	-0.0138	7.7E-04	77326662	rs483810	G: 0.83/0.39	Intergenic	-0.0061	0.04
9	77.513–77.552	rs17060609	rs2376378	*TRPM6 | C9orf40*	-0.0137	8.0E-04	77516005	rs11144144	A: 0.56/0.28	Intergenic	-0.0076	0.01
6	53.674–53.78	rs4269375	rs10402616	*LRRC1*	0.0138	8.4E-04	53689508	rs6907027	A: 0.34/0.26	Intron	-0.0075	0.02
19	53.763–53.769	rs7256218	rs16984664	*VN1R2 | VN1R4*	0.0138	8.4E-04	‒	‒	‒	‒	‒	‒
**HIV-negative**												
10	121.711–122.215	rs10749330	rs2463147	*SEC23IP | PPAPDC1A*	-0.0290	1.7E-05	122197661	rs2463128	C:0.01/0.31	Intergenic	0.0168	5.1E-04
10	122.358–122.358	rs2901257	rs2901257	*C10orf85*	-0.0272	5.8E-05	‒	‒	‒	‒	‒	‒
10	122.365–122.601	rs11199437	rs12220751	*C10orf85 | BRWD2*	-0.0272	5.8E-05	122480687	rs4751799	C: 0.22/0.55	Intergenic	-0.0162	3.4E-04*
10	120.522–120.784	rs10886296	rs494469	*C10orf46 | NANOS1*	-0.0266	7.4E-05	120742200	rs10886356	T: 0.15/0.18	Intergenic	-0.0174	0.008
10	121.218–121.257	rs10886486	rs10787973	*GRK5 | RGS10*	-0.0269	8.0E-05	121251611	rs11198973	G: 0.28/0.48	Intergenic	-0.0124	0.007

SNP: single nucleotide polymorphism.

*Significant (*P*<0.05) after adjusting for number of SNPs in each region.

### Meta-analysis and previously significant SNPs

Previously significant SNPs associated with cCIMT that were found significant in the current GWAS and LEA association analysis are reported in the **Tables 4 and 5**, respectively. A total of 25 SNPs in the GWAS and 31 SNPs in the admixture analysis were significantly associated with cCIMT. The most significant SNPs across the combined and stratified GWAS analyses were—rs445925 in the apolipoprotein C-1 (*APOC1*) gene among all women (β = -0.0081, p = 0.0018), rs2572204 in the ryanodine receptor 3 (*RYR3*) among HIV-positive women (β = 0.0153, p = 0.0027), and rs854571 in paraoxonase 1 (*PON1*) gene in the HIV-negative women (β = 0.0107, p = 0.02). The most significant SNPs in the LEA association included rs1436023 in *LOC105375856* gene in the combined analysis (β = -0.0099, p = 0.005), the region between 17.809 to 18.035 Mb in the *SERGEF* gene significantly associated with cCIMT among the HIV-positive women (β = -0.0093, p = 0.02) whereas the SNP rs11120748 in the CD55 molecule (Cromer blood groups) (*CD55*) gene was significant among HIV-positive women (β = -0.0156, p = 0.02).

**Table 4:** Genome wide association results (GWA) for common carotid artery intima media thickness with previously significant single nucleotide polymorphisms (SNPs).

Chromosome	Position	SNP	Minor allele	Gene	Location	Beta	P from GWAS
Combined							
19	45415640	rs445925	T	*APOC1*		-0.0081	0.002
7	68047178	rs10248387	G	*LOC102723427*, *LOC100507468*		-0.0074	0.006
9	106487753	rs4436177	G	*LINC01492*, *LOC101928523*		0.0099	0.02
15	33795710	rs2572204	A	*RYR3*		0.0098	0.02
7	94954619	rs854571	C	*PON1*		0.0058	0.02
15	31421676	rs783026	G	*TRPM1*		0.0057	0.02
5	149176945	rs1012543	C	*PPARGC1B*		0.0083	0.03
18	8797189	rs566890	G	*MTCL1*		-0.0051	0.03
2	239128117	rs6722019	T	*ILKAP*, *LOC151174*		0.0051	0.04
13	106376128	rs1328070	T	*LINC00343*		0.0074	0.04
6	135237158	rs6569979	G	*HBS1L*		0.0049	0.04
HIV-positive							
15	33795710	rs2572204	A	*RYR3*		0.0153	0.003
7	68047178	rs10248387	G	*LOC102723427*, *LOC100507468*		-0.0095	0.004
9	106487753	rs4436177	G	*LINC01492*, *LOC101928523*		0.0139	0.005
19	45415640	rs445925	T	*APOC1*		-0.0079	0.01
15	98680170	rs2871596	C	*LINC01582*, *FAM169B*		-0.0067	0.03
HIV-negative							
7	94954619	rs854571	C	*PON1*		0.0107	0.02
20	56157341	rs6025645	A	*PCK1*		-0.0105	0.02
4	2906707	rs4961	T	*ADD1*		0.0189	0.02
18	8797189	rs566890	G	*MTCL1*		-0.0096	0.02
4	75683594	rs4352548	C	*BTC*		0.0209	0.03
10	45917376	rs3780901	C	*ALOX5*		0.0098	0.03
7	33701425	rs10246872	G	*BBS9*, *BMPER*		0.0091	0.04
1	92076523	rs1041159	C	*CDC7*, *TGFBR3*		-0.0088	0.04
5	13214852	rs28207	T	*LOC105374659*, *RPS23P5*		-0.0131	0.04

**Table 5:** Admixture (LEA) results for common carotid-artery intima media thickness association with previously significant single nucleotide polymorphisms (SNPs).

Chromosome	Position	SNP	Gene	Beta	P from GWAS
Combined					
8	58843783	rs1436023	*LOC105375856*	-0.0099	0.005
8	56847170	rs13249338	*LYN*	-0.0099	0.005
2	98017804	rs11901495	*LOC107985919*	0.0096	0.005
8	55340313	rs13272749	*LOC105375841*	-0.0096	0.006
8	55327862	rs6987174	*MRPL15*, *SOX17*	-0.0096	0.006
8	55318559	rs7834421	*MRPL15*, *SOX17*	-0.0096	0.006
8	55369597	rs12234926	*SOX17*	-0.0095	0.007
5	4241397	rs7702251	*IRX1*, *LOC101929153*	0.0078	0.02
8	42045655	rs2070713	*PLAT*	-0.0071	0.04
HIV-positive					
11	17.809–18.035	rs1236207- rs6486402	*SERGEF*	-0.0093	0.02
5	4241397	rs7702251	*IRX1*, *LOC101929153*	0.0097	0.02
9	74974635	rs11143274	*ZFAND5*	-0.0091	0.02
8	58843783	rs1436023	*LOC105375856*	-0.0094	0.03
7	151255751	rs4726047	*PRKAG2*	-0.0089	0.03
1	102423144	rs4907957	*OLFM3*	0.0098	0.03
9	7815105	rs1360583	*TMEM261*, *PTPRD*	0.0090	0.03
19	45415640	rs445925	*APOC1*	0.0083	0.03
19	45814860	rs7260463	*CKM*	0.0081	0.04
19	35052255	rs2199639	*WTIP*, *SCGB1B2P*	0.0086	0.04
19	45740771	rs17356664	*EXOC3L2*	0.0080	0.04
2	98017804	rs11901495	*LOC107985919*	0.0082	0.04
HIV-negative					
1	207524541	rs11120748	*CD55*	-0.0156	0.02
8	56847170	rs13249338	*LYN*	-0.0148	0.03
2	98017804	rs11901495	*LOC107985919*	0.0132	0.03
9	71316454	rs1412990	*LINC01506*, *PIP5K1B*	0.01285	0.04
8	55369597	rs12234926	*SOX17*	-0.0141	0.04
8	55340313	rs13272749	*LOC105375841*	-0.0141	0.04
8	55327862	rs6987174	*MRPL15*, *SOX17*	-0.0141	0.04
8	55318559	rs7834421	*MRPL15*, *SOX17*	-0.0141	0.04
18	75938925	rs1587893	*LOC10042152*, *LOC645321*	0.0120	0.04
12	63178779	rs2641558	*PPM1H*	-0.0126	0.04
12	63174735	rs337515	*PPM1H*	-0.0126	0.04

Meta-analysis results for both GWAS and LEA associations reflect the results observed in the combined analyses (**S1 and S2 Tables**).

## Discussion

The current study presents the genome-wide admixture and association findings for cCIMT among Black HIV-positive and HIV-negative women from the WIHS cohort. We ran separate analyses for the two methods but also noted whether any of the GWAS SNPs within the top admixture regions, and any of the admixture regions including the top GWAS SNPs were significant for cCIMT. We observed SNPs in the GWA that showed suggestive associations (p<10^−6^) with cCIMT (included in **Table 2** in bold). The comparison to the admixture results yielded two significant regions observed among HIV-positive women (included in **Table 2** in bold). Conversely, several regions with LEA associations revealed GWAS SNPs that were nominally significant. Meta-analysis results suggest that some SNPs and gene regions are common across HIV-positive and HIV-negative women and may therefore play a role in atherosclerosis beyond HIV associated risk factors. Lastly, we observed associations with SNPs that have been previously reported to have significant associations with cCIMT.

The top GWAS hit rs2280828 observed in the combined analysis was in the region intergenic to the *MED30* and *EXT1* genes on chromosome 8, approximately 50 kb from the *EXT1* gene. The *EXT1* gene encodes for a transmembrane glycoprotein on the endoplasmic reticulum (ER) of the cell which is involved in the synthesis and polymerization of heparan sulfate [33, 34]. Heterozygous loss of function mutation in the *EXT1* and *EXT2* genes have been postulated to improve endothelial function by increasing the bioavailability of nitric oxide (NO) [35]. Calcium ion concentrations in the cell regulate the activity of nitric oxide synthase via calmodulin, indirectly influencing the release of nitric oxide when shear stress is exerted on the endothelium [36]. The RYR3 receptor encoded by the *RYR3* gene is a calcium-ion channel present on the ER and is involved in calcium homeostasis [37]. Thus, *EXT1* may play a role in atherosclerosis by regulating endothelial function in conjunction with *RYR3*. On the other hand, the *MED30* gene is part of a large group of genes that encode the mediator complex proteins, constituting a multi-subunit complex, known to regulate the RNA polymerase II enzyme [38]. Silencing of this gene was shown to inhibit the transcription of HIV, in part via the HIV-Tat protein [38]. The HIV-Tat protein causes endothelial dysfunction and brings about an inflammatory response as well as triggers loss of calcium through the RYRs on the ER [39]. These factors are part of the biological mechanisms that could contribute to atherosclerosis in HIV. Therefore, *MED30* gene-related functions may play a role in mitigating the downstream effects of HIV in atherosclerosis and other cardiovascular complications. Spliced variants of the gene have been observed in circulating endothelial progenitor cells and are hypothesized to have potential diagnostic or therapeutic roles in cardiovascular disease in the future [40].

Rs2907092 was the top GWAS hit among HIV-positive women and is located in the intronic region of the *CTNND2* gene on chromosome 5. This gene encodes for the δ–catenin protein, a neuronal protein that is also expressed in the vascular endothelium [41]. The protein binds cadherins to the cytoskeleton and is involved in pathways and mechanisms that modulate vascular remodeling. They are responsible for endothelial cell motility, angiogenesis and wound repair regulated by inflammatory responses and are specifically observed during pathologic states. This gene showed a strong association with cerebral malaria in an environmental correlation analysis (ECA), whereby the involved mechanisms such as luminal blockade by infected cells, increased shear stress, presence of reactive oxygen species (ROS) and the ensuing inflammatory response follow a pattern that is similar to atherosclerosis [42]. ECA identifies genes that are enriched as a result of local environmental effects within a population that has likely been isolated for a long period of time. As a result, the *CTNND2* gene may likely have an admixture component, which was precisely noted in our admixture results.

The most significant GWAS SNP among HIV-negative women was rs7529733 in the regions intergenic to the *FAM5C* and *RGS18* genes on chromosome 1. The *FAM5C* gene has been associated with myocardial infarction [43] and functional studies have shown that it increases the production of ROS, nuclear factor-kappaB (NF-κB) activity, and expression of the intercellular and vascular cell adhesion molecules, ICAM-1 and VCAM-1 [44]. These factors induce vascular inflammation and facilitate monocyte adhesion to the endothelium. HIV proteins such as HIV-Tat trigger this mechanism indirectly by activating the monocyte chemoattractant protein-1 (MCP-1) resulting in migration of the monocytes and their transformation into macrophages, steps integral to the atherosclerotic process [39]. The regulator of G-protein signaling 18 GTPase-activating protein is encoded by the *RGS18* gene. This protein is involved in the activation and inhibition of platelets through the G-α-q subunit of the heterotrimeric G-protein [45]. The RGS18 protein is in turn regulated by thrombin resulting in the phosphorylation of one of its residues, and subsequent release of calcium ions to induce platelet aggregation. A similar process is involved during platelet inhibition whereby prostacyclin and NO regulate RGS18 resulting in reduced intracellular calcium levels. Thus, the RGS18 effect on platelets is mediated through calcium homeostasis and therefore genetic interactions between *RGS18* and *RYR3* gene are possible, and may play a substantial role in atherosclerosis as platelets are involved in its inflammatory response.

The top admixture mapping results for the combined, and stratified analyses among HIV-positive women revealed genes that are pseudogenes, and which have not yet been evaluated for associations with human complex diseases or traits. The second top hit involves the galanin-like peptide encoded by the *GALP* gene on chromosome 19. It is a neuropeptide of the galanin family and has been shown to play a role in homeostatic processes of the central nervous system (CNS) [46]. These include but are not limited to hormonal regulation of food intake with the potential to develop obesity and other weight gain related complications like diabetes. Additionally, a splice variant of the peptide called alarin was observed to have vasoactive properties and induced vasoconstriction and decrease in water retention in the epidermis. Diabetes and obesity are risk factors for atherosclerosis and CVD, and vascular perturbations in the microvasculature are known to induce downstream effects that play a role in the atherosclerotic process. The *TRPM6* gene located on chromosome 9 was associated with cCIMT among HIV-positive women. This gene encodes for the transient receptor potential melastatin 6 channel which acts as both a channel and an enzyme involved in the regulation of magnesium (Mg^2+^) in the body [47]. TRPM6 and TRPM7 were both found in the vascular smooth muscle cells and were shown to regulate hypertension via Mg^2+^ homeostasis. Reduced Mg^2+^ increases blood pressure by increasing the reactivity and contractility of the blood vessels and is also responsible for endothelial dysfunction, inflammation and vascular remodeling. All the above-mentioned mechanisms are involved in the ongoing process of atherosclerosis and subsequent CVD and therefore TRPM6 may play a part in the development of CVD. Among HIV-negative women, the top LEA region was on chromosome 10 and located in the intergenic region of the *SEC23IP* and *PPAPDC1A* genes. The rs1571099 SNP in *PPAPDC1A* was significantly associated with interventricular septal wall thickness among African Americans in a recent GWAS using the Candidate Gene Association Resource (CARe) Study [48]. This gene is involved in pathways related to innate immunity, phagocytosis and triacylglycerol synthesis [49]. Therefore, this gene may also play a role in atherosclerosis, albeit an indirect one.

Replication revealed associations with some of the cCIMT-SNPs found significant in earlier studies. Previously, we had reported the association of two SNPs in *RYR3* –rs2229116 and rs7177922 that had achieved genome-wide significance among HIV-positive men of European ancestry [20], with successful replication of rs2229116 in another group of HIV-positive White men [21]. However, we did not find any association with these SNPs in the current analysis. Instead, we found another SNP–rs2572204 in *RYR3* that was associated with cCIMT among HIV-positive women of WIHS. We reported the same association among Black HIV-positive women in our previous candidate-gene study on *RYR3* in WIHS. The different SNP associations within this gene may either be because of the differences in race or sex between the two studies. WIHS women are also noted to have different heart disease risk because of differences in their immunosuppression or duration of ART as compared to HIV-positive men [50, 51]. Nonetheless, *RYR3* appears to play a role in HIV-related atherosclerosis risk based on some of the possible gene (or protein) interactions found significant in this study. The admixture association of the *SERGEF* gene in the HIV-positive women provides further support for the role of this gene in atherosclerosis. However, the direction of association was opposite to that observed in the HIV-negative population in our previous study. The gene encodes a guanine nucleotide exchange factor that may play a role in atherosclerosis through immune or inflammatory mechanisms via guanosine-triphosphatases and serotonin [25]. We speculate that lower cCIMT associated with *SERGEF* in the HIV-positive women may be a result of immunosuppression because of ART or HIV itself or in conjunction with the gene as it appears to mediate atherosclerosis through immune-related mechanisms.

Population stratification is an issue that needs to be addressed during GWAS to avoid spurious associations, more so in admixed populations. Literature on genetic association studies among admixed populations has persistently examined different approaches to account for population structure. An initial and basic approach was genomic control [52] which was improved upon by Price et al. [27] who suggested controlling for principal components. This is currently the mostly widely used approach in GWAS and other genetic association studies among admixed populations. However, recent studies argue that adjusting for global ancestry is not sufficient and does not account for ancestry at the marker level for each individual SNP which is commonly referred to as local ancestry [28]. To this effect, joint testing of admixture and association was proposed and demonstrated an increase in statistical power and accuracy of the results compared to existing methods [30, 31]. In the current study, we compared the performance of two different methods based on level of ancestry adjustment; the methods are described in the supplementary methods section (**S1 File**) and the results are presented in **S3 Table**. The comparison shows that the power is highest for the QSUM test that adjusts for both local and global ancestries using the MIXSCORE program. Pasaniuc et al. reported that the QATT test performed better for quantitative traits but also noted that the QSUM test might perform better given a set of conditions where high signals of admixture associations were observed [30]. The BMIX test as proposed by Shriner et al. performs even better than the QSUM test with a power of ~72% to detect phenotype-genotype associations in the overall analysis [31]. Both results support the hypothesis that joint testing with adjustment for both local and global ancestries is more powerful than the current association tests in use. Therefore, we performed a secondary analysis using BMIX and present our results in the supplemental **S4 Table**. Of note, the top SNPs in Table 2 showed similar significance after adjustment for local ancestry using BMIX (if available). Since BMIX uses the local ancestry information available through admixture mapping, as such the two analyses are not independent of each other. In our study, we did not conduct the primary GWAS analyses using the BMIX method as we could only estimate local ancestry in 473,732 SNPs, and so adjustment with local ancestry would have dramatically reduced the number of informative SNPs available for GWAS. Nevertheless, this approach of adjustment for both local and global ancestry has not been extensively explored and needs further validation to ensure broader application in future genetic studies among admixed populations and also tease shared traits across ancestries [53].

The advantage of using the WIHS cohort is that a higher proportion of women in the cohort were African American, especially among the HIV-positive subpopulation. It also presented us with an opportunity to evaluate the common CIMT association exclusively among women. However, we recognize that the homogeneity of the population also reduces the generalizability of our findings. As the WIHS participants are relatively young and the incidence of clinical CVD events is low, we could not assess the association of CVD events at the genome-wide level. Therefore, we assessed cCIMT which is a reliable and reproducible subclinical measure of atherosclerosis that has been shown to predict future CVD events. Most of the HIV-positive women were also on combination ART including multiple antiretroviral drugs which prevented us from evaluating the independent effect of specific ART agents on cCIMT. Furthermore, our admixture analysis only included a subset of the SNPs that were part of the GWAS (N = 473,732), and so we were limited in our ability to assess local ancestry associations at the excluded SNPs. This also limited our ability to adjust for both local and global ancestry during GWAS using the joint admixture and association testing method. We acknowledge that the sample size was small for a genome-wide analysis, however, WIHS is currently the largest prospective cohort of HIV-positive women in the U.S. with the cCIMT measurements since cCIMT is not readily available, specifically among HIV-positive women. The heterogeneity index I^2^ (**S1 and S2 Tables**) also shows that there are some consistent results (both in direction of association and significance) but also inconsistent ones between HIV-positive and HIV-negative groups. These results may suggest differences in underlying biological mechanism, i.e. some of the genes or pathways maybe specific in the context of HIV (virus itself or treatment) and others similar in both with or without infection, or they could be artifacts as a result of the small sample size. While we cannot conclude the reason of heterogeneity in the two groups, the suggestive results should be examined in other populations.

In summary, we report several novel associations in relation to common CIMT among a group of Black HIV-positive and HIV-negative women observed through genome-wide association and admixture analysis. The results suggest that GWAS results do not completely capture associations in regions of local ancestry and vice-a-versa among admixed populations and both approaches may still be necessary to evaluate genotype-phenotype associations in specific settings. These findings need to be confirmed in larger studies with local ancestry available for a denser set of markers to allow equal comparisons across GWAS and admixture results. Future studies may also benefit from combining data from other HIV cohorts and performing a meta-analysis to detect novel or stronger associations.

## Methods

### Ethics statement

The parent WIHS study and this sub-study conformed to the procedures for informed written consent approved by institutional review boards (IRB) at all sponsoring organizations and to human-experimentation guidelines set forth by the United States Department of Health and Human Services, and finally reviewed and approved by the University of Alabama Institute Review Board.

### Study population

WIHS is a large prospective cohort of HIV-positive and risk-matched HIV-negative women enrolled across six sites in the United States [54, 55]. The women in the study were enrolled during the initiation phase in 1994/95 (2054 HIV-positive and 569 HIV-negative), the expansion phase in 2001/02 (737 HIV-positive and 406 HIV-negative), and most recently during 2011/2012 to replace the women who had died during follow-up (270 HIV-positive and 93 HIV-negative). The study was designed to comprehensively evaluate the impact of HIV infection in U.S. women, and provide comparisons with the HIV-negative group having similar characteristics and a high risk of acquiring the infection themselves. The women in the expansion phase were included to examine the risks and benefits associated with ART. A vascular disease sub-study was initiated in April 2004 to examine the effect of HIV and ART on subclinical atherosclerosis over time, focusing specifically on inflammation, coagulation and lipid pathways [50]. The measurements for cCIMT were obtained among 1331 HIV-positive and 534 HIV-negative women in WIHS through the vascular sub-study. In the current study, 970 Black, non-Hispanic women (682 HIV-positive and 288 HIV-negative) with complete genotype, phenotype and covariate information were included, after confirming the race/ethnicity for these women using the principal components method.

#### Data collection

The collection and processing of data in WIHS was standardized with centralized training of all staff before initiation of enrollment. Interviews with structured questionnaires were conducted to obtain participant-reported information on demographic, lifestyle, socio-economic and clinical factors. In addition, anthropometric measures such as weight and height; physiologic measures such as blood pressure (BP); laboratory measures such as markers of HIV, and lipid profile were obtained using standardized protocols; and gynecologic examinations were conducted. Follow-up every six months included collection of interview related information and medication use [54].

CIMT images were obtained via high-resolution ultrasound by sonographers trained centrally at the University of Southern California Atherosclerosis Research Unit Core Imaging and Reading Center using a standard protocol across all study sites [56]. CIMT was measured by the automated computerized edge-tracking method (patents 2005, 2006, 2011) [57]. The coefficient of variation was 1.8% (intraclass correlation = 0.98; n = 113) for repeated CIMT measurements with the initial images guiding the repeat scans. The complete ultrasound included: 1) standardized measurements of the far-right wall of the distal common carotid artery for CIMT, stiffness and lesions, and 2) scanning the proximal internal carotid, external carotid and right carotid bulb at the bifurcation for lesions [50]. In the current study, we utilized the common carotid artery measurements from the distal far right wall. The mean right wall cCIMT was log base 10 transformed for normalization.

#### Genotyping

DNA isolation, quantification and the microplate reading were performed using the Pure-gene DNA isolation kit (Gentra Systems, Minneapolis, MN), the PICO Green dsDNA quantification kit (Molecular Probes, Eugene, OR) and the Perkin Elmer HTS7000 BioAssay Reader, respectively [58]. All samples were stored at -20 ^o^C. The genome-wide scan was performed using the Illumina HumanOmni2.5-quad beadchip (NCBI build 37, hg19, Illumina, San Diego).

### Statistical analysis

Both the genome-wide and ancestry association analyses were initially conducted for all individuals followed by stratified analyses among HIV-positive and HIV-negative Black women. Common CIMT was associated with all CVD risk factors except HDL cholesterol, and was also not associated with ART or CD4 cell count in bivariate analysis. However, HIV related risk factors such as ART and CD4 cell counts have been previously associated with cCIMT and were therefore included in the final analysis.[11, 50] Analyses among all and the HIV-positive individuals were adjusted for age, CD4 cell count, ART use, hypertension (systolic BP≥140 mmHg, diastolic BP≥90 mmHg, antihypertensive medication use or self-report), diabetes mellitus (anti-diabetic medication use, fasting glucose ≥126mg/dl, HgbA1C≥6.5%, or self-report), current smoking, low-density lipoprotein (LDL), and high-density lipoprotein (HDL) cholesterol. Covariate adjustment for HIV-negative individuals was the same except for ART use.

#### Quality control of genomic data and GWA analysis

Multivariable linear regression analysis using an additive model was performed in PLINK (v1.9) with default settings [59]. In the current study, genotype information for a total of 1,590,141 SNPs was available. Quality control resulted in the removal of 2 SNPs because of missingness at >10%, 6,433 because of Hardy-Weinberg equilibrium <0.001, and 459,405 because of minor allele frequency (MAF) <5%, leaving a total of 1,124,301 SNPs to be included in the genome-wide association analysis (**S5 Table**). Genetic ancestry components were evaluated with principal component (PC) analysis using 168 ancestry informative markers (AIMs) as previously described [60]. Briefly, the number of PCs which distinguished the major racial/ethnic groups in the sample was sought by visual inspection of scatter plots of orthogonal PCs (i.e., PC 1 versus PC2, PC2 versus PC3). This procedure was repeated until no discernible clustering of individuals by their self-reported race/ ethnicity was possible (data not shown) [60, 61]. Based on the scree plot (**S2 Fig**), GWAS analysis was adjusted for 2 principal components (PCs) in addition to the other covariates, described above. We also tested for genomic inflation of the GWAS results by obtaining the lambda values as well as QQ plots for both the combined as well as stratified models. Correction for multiple testing was performed using a method proposed by Shriner et al [31]. The method estimates the effective number of tests by fitting an autoregressive model to the additively coded genotypes and choosing the order of the model based on the lowest Akaike information criteria (AIC). The total number of independent tests estimated using this method were 769,204, resulting in a threshold significance level of 6.50 x 10^−8^.

#### Local ancestry estimation

Local ancestry was estimated using the Local Ancestry in adMixed Populations using Linkage Disequilibrium (LAMP-LD) program [62]. The program incorporates a dense set of markers to determine ancestry at each SNP for each individual based on haplotype sets from ancestral populations. Phased haplotype data for the reference populations was obtained from HapMap phase II and III data from Utah residents with North-West European ancestry (CEU) and Yorubans from Ibadan, Nigeria (YRI) for African ancestry (HapMap phase 3, release 2). The reference haplotype data was used to determine parameters for the Hidden Markov Model (HMM) with 15 state spaces. The local ancestry was estimated using the HMM parameters within a 300 SNP-long window-based framework. Each chromosome was analyzed separately and the local ancestry coded as the number of European ancestry alleles at each SNP (i.e., 0, 1, or 2 where “0” means both chromosomal regions had African ancestry, “1” means the chromosomal region is admixed, one with European ancestry and the other with African ancestry, and “2” means both chromosomal regions had European ancestry). Before ancestry estimation, the physical coordinates for the remaining 1,124,301 SNPs were converted from hg19 to hg18 build to match the coordinates with the HapMap phased data using the UCSC table browser [63]. As a result, 119 SNPs were lost during conversion. Furthermore, 648,814 SNPs were not available in HapMap, 1,259 had ambiguous A/T or G/C SNPs with MAF >35% and could not be resolved for strand annotation, and 63 were duplicates; this resulted in 473,732 autosomal SNPs remaining for inclusion in the admixture estimation and association analyses (**S5 Table**).

A SNP most significantly associated with the clinical events along with the block of neighboring SNPs with similar number of European ancestry (“0”, “1” or “2”) were denoted as a “LEA region”. Additionally, blocks of same ancestry LEA regions were further defined based on whether the SNPs were in the genic or intergenic regions. A genic region included the 5’ and 3’ untranslated regions, the exons and the introns; the intergenic regions were those that fell between two genic regions as described above. Gene nomenclatures were obtained from the National Center for Biotechnology Information’s (NCBI) Reference Sequence database through the web ANNOVAR program [64].

#### Local ancestry association with cCIMT

Association testing in relation to cCIMT was performed using the PLINK (v1.9) software with default settings [59]. We conducted an additive linear regression analysis to evaluate the association of local ancestry coded as homozygous European ancestry (11), heterozygous European/African ancestry (12) and homozygous African ancestry (22), controlling for global European ancestry in the model. Since ancestry estimates are highly correlated, it is necessary to calculate the total number of effective independent tests to arrive at a threshold genome-wide significance level. We calculated the total number of effective independent tests using the method proposed by Shriner et al for admixture analysis as well [31, 65]. The total number of independent tests in our analyses was 146 resulting in a threshold significance level of 3.42 x 10^−4^.

#### Meta-analysis

Meta-analysis was performed on the results from the GWAS and admixture analyses separately. The coefficients obtained from the stratified analyses of HIV-positive and HIV-negative women were combined to obtain a pooled estimate. The analysis was conducted using PLINK (v1.9) for quantitative traits with both fixed- and random-effects analysis, including the detection of heterogeneity of the results (between HIV-positive and HIV-negative) using the heterogeneity index I^2^ (**S1 and S2 Tables**).

### Evaluation of previously significant cCIMT SNPs

We identified 377 SNPs based on previous cCIMT-genetic association studies (**S6 Table**). Of these, 145 SNPs were obtained from a combination of candidate gene, genome-wide (GWAS) or meta-analysis studies [16, 19, 20, 22, 66–87], 232 SNPs from a GWAS conducted by Melton et. al. [17]. We assessed the GWAS results and LEA regions that contained these SNPs and report the associations for those found significant at P<0.05 in our cCIMT analysis.

## Supporting information

S1 File Supplemental methods. Comparing test performance after ancestry adjustment.
(DOCX)

S1 Table Genome-wide association (GWA) results for cCIMT in the combined, HIV-positive, and HIV-negative models, including the meta-analysis results. (XLSX)

S2 Table Genome-wide admixture (LEA) results for cCIMT in the combined, HIV-positive, HIV-negative models, including the meta-analysis results. (XLSX)

S3 Table Comparing test performance after adjusting for global, local or both ancestries in the association tests. QATT: quantitative Armitage’s trend test adjusting for global ancestry, QSNP1: quantitative single nucleotide polymorphism association adjusting for local ancestry, QADM: quantitative admixture test, QSUM: sum of QSNP1 and QADM, BMIX: joint admixture and association test using a Bayesian approach, Score: minus log 10 p-values.
(DOCX)

S4 Table Joint association and admixture (LEA) results for cCIMT in the combined, HIV-positive, and HIV-negative models using the BMIX method. (XLSX)

S5 Table Quality control of the genome wide data from the Women’s Interagency HIV study (WIHS). SNP: single nucleotide polymorphism, MAF: minor allele frequency.
(DOCX)

S6 Table List of SNPs previously significantly associated with cCIMT in candidate gene, genome-wide association or meta-analysis studies*. *Publications before the year 2004 are represented in two reviews by Manolio TA 2004 and Pollex RL 2006. Two or more publications are referenced for some SNPs. Several SNP or gene associations were common across studies but are only presented once in the table. Publications in bold were the only studies that included a HIV-positive population.
(XLSX)

S1 Fig Regional plots for the topmost hit from the genome-wide association and admixture analyses. a) All women (GWAS), b) HIV-positive women (GWAS), c) HIV-negative women (GWAS), d) All women (admixture), e) HIV-positive women (admixture), and f) HIV-negative women (admixture). Negative log10 p-values and recombination rates are plotted against SNPs and their chromosomal positions.
(PDF)

S2 Fig Scree plot showing the eigenvalues plotted against the principal components. (PDF)

## References

[1] Farnham PG, Gopalappa C, Sansom SL, Hutchinson AB, Brooks JT, Weidle PJ, et al Updates of lifetime costs of care and quality-of-life estimates for HIV-infected persons in the United States: late versus early diagnosis and entry into care. J Acquir Immune Defic Syndr. 2013;64(2):183–9. Epub 2013/04/26. doi: 10.1097/QAI.0b013e3182973966 . 23615000 10.1097/QAI.0b013e3182973966

[2] Triant VA. Cardiovascular Disease and HIV Infection. Curr HIV/AIDS Rep. 2013 doi: 10.1007/s11904-013-0168-6 . 23793823 10.1007/s11904-013-0168-6 PMC3964878

[3] Triant VA, Lee H, Hadigan C, Grinspoon SK. Increased acute myocardial infarction rates and cardiovascular risk factors among patients with human immunodeficiency virus disease. J Clin Endocrinol Metab. 2007;92(7):2506–12. doi: 10.1210/jc.2006-2190 ; PubMed Central PMCID: PMCPMC2763385. 17456578 10.1210/jc.2006-2190 PMC2763385

[4] Lang S, Mary-Krause M, Cotte L, Gilquin J, Partisani M, Simon A, et al Increased risk of myocardial infarction in HIV-infected patients in France, relative to the general population. AIDS. 2010;24(8):1228–30. doi: 10.1097/QAD.0b013e328339192f . 20400883 10.1097/QAD.0b013e328339192f

[5] Oramasionwu CU, Hunter JM, Brown CM, Morse GD, Lawson KA, Koeller JM, et al Cardiovascular Disease in Blacks with HIV/AIDS in the United States: A Systematic Review of the Literature. Open AIDS J. 2012;6:29–35. Epub 2012/05/09. doi: 10.2174/1874613601206010029 ; PubMed Central PMCID: PMCPmc3343316. 22563364 10.2174/1874613601206010029 PMC3343316

[6] Oramasionwu CU, Morse GD, Lawson KA, Brown CM, Koeller JM, Frei CR. Hospitalizations for cardiovascular disease in African Americans and whites with HIV/AIDS. Popul Health Manag. 2013;16(3):201–7. Epub 2012/12/01. doi: 10.1089/pop.2012.0043 ; PubMed Central PMCID: PMCPmc3840471. 23194035 10.1089/pop.2012.0043 PMC3840471

[7] Chow FC, Regan S, Feske S, Meigs JB, Grinspoon SK, Triant VA. Comparison of ischemic stroke incidence in HIV-infected and non-HIV-infected patients in a US health care system. J Acquir Immune Defic Syndr. 2012;60(4):351–8. doi: 10.1097/QAI.0b013e31825c7f24 ; PubMed Central PMCID: PMC3670086. 22580566 10.1097/QAI.0b013e31825c7f24 PMC3670086

[8] Lorenz MW, Markus HS, Bots ML, Rosvall M, Sitzer M. Prediction of clinical cardiovascular events with carotid intima-media thickness: a systematic review and meta-analysis. Circulation. 2007;115(4):459–67. doi: 10.1161/CIRCULATIONAHA.106.628875 . 17242284 10.1161/CIRCULATIONAHA.106.628875

[9] Kanters SDJM, Algra A, Van Leeuwen MS, Banga JD. Reproducibility of in vivo carotid intima-media thickness measurements: A review. Stroke. 1997;28(3):665–71. 9056629 10.1161/01.str.28.3.665

[10] Nambi V, Chambless L, He M, Folsom AR, Mosley T, Boerwinkle E, et al Common carotid artery intima-media thickness is as good as carotid intima-media thickness of all carotid artery segments in improving prediction of coronary heart disease risk in the Atherosclerosis Risk in Communities (ARIC) study. Eur Heart J. 2012;33(2):183–90. doi: 10.1093/eurheartj/ehr192 ; PubMed Central PMCID: PMC3258447. 21666250 10.1093/eurheartj/ehr192 PMC3258447

[11] Lorenz MW, Stephan C, Harmjanz A, Staszewski S, Buehler A, Bickel M, et al Both long-term HIV infection and highly active antiretroviral therapy are independent risk factors for early carotid atherosclerosis. Atherosclerosis. 2008;196(2):720–6. Epub 2007/02/06. doi: 10.1016/j.atherosclerosis.2006.12.022 . 17275008 10.1016/j.atherosclerosis.2006.12.022

[12] Hsue PY, Lo JC, Franklin A, Bolger AF, Martin JN, Deeks SG, et al Progression of atherosclerosis as assessed by carotid intima-media thickness in patients with HIV infection. Circulation. 2004;109(13):1603–8. Epub 2004/03/17. doi: 10.1161/01.CIR.0000124480.32233.8A . 15023877 10.1161/01.CIR.0000124480.32233.8A

[13] Stein JH, Brown TT, Ribaudo HJ, Chen Y, Yan M, Lauer-Brodell E, et al Ultrasonographic measures of cardiovascular disease risk in antiretroviral treatment-naive individuals with HIV infection. AIDS. 2013;27(6):929–37. Epub 2012/12/01. doi: 10.1097/QAD.0b013e32835ce27e ; PubMed Central PMCID: PMCPmc3664137. 23196938 10.1097/QAD.0b013e32835ce27e PMC3664137

[14] Delaney JA, Scherzer R, Biggs ML, Shliplak MG, Polak JF, Currier JS, et al Associations of antiretroviral drug use and HIV-specific risk factors with carotid intima-media thickness. AIDS. 2010;24(14):2201–9. Epub 2010/07/31. doi: 10.1097/QAD.0b013e32833d2132 ; PubMed Central PMCID: PMCPmc3224487. 20671544 10.1097/QAD.0b013e32833d2132 PMC3224487

[15] Grunfeld C, Delaney JA, Wanke C, Currier JS, Scherzer R, Biggs ML, et al Preclinical atherosclerosis due to HIV infection: carotid intima-medial thickness measurements from the FRAM study. AIDS. 2009;23(14):1841–9. doi: 10.1097/QAD.0b013e32832d3b85 ; PubMed Central PMCID: PMCPMC3156613. 19455012 10.1097/QAD.0b013e32832d3b85 PMC3156613

[16] Bis JC, Kavousi M, Franceschini N, Isaacs A, Abecasis GR, Schminke U, et al Meta-analysis of genome-wide association studies from the CHARGE consortium identifies common variants associated with carotid intima media thickness and plaque. Nat Genet. 2011;43(10):940–7. Epub 2011/09/13. doi: 10.1038/ng.920 ; PubMed Central PMCID: PMCPmc3257519. 21909108 10.1038/ng.920 PMC3257519

[17] Melton PE, Carless MA, Curran JE, Dyer TD, Goring HH, Kent JW Jr., et al Genetic architecture of carotid artery intima-media thickness in Mexican Americans. Circ Cardiovasc Genet. 2013;6(2):211–21. Epub 2013/03/15. doi: 10.1161/CIRCGENETICS.113.000079 ; PubMed Central PMCID: PMCPmc3865281. 23487405 10.1161/CIRCGENETICS.113.000079 PMC3865281

[18] Wang D, Yang H, Quinones MJ, Bulnes-Enriquez I, Jimenez X, De La Rosa R, et al A genome-wide scan for carotid artery intima-media thickness: the Mexican-American Coronary Artery Disease family study. Stroke. 2005;36(3):540–5. doi: 10.1161/01.STR.0000155746.65185.4e . 15692111 10.1161/01.STR.0000155746.65185.4e

[19] Natarajan P, Bis JC, Bielak LF, Cox AJ, Dorr M, Feitosa MF, et al Multiethnic Exome-Wide Association Study of Subclinical Atherosclerosis. Circ Cardiovasc Genet. 2016;9(6):511–20. doi: 10.1161/CIRCGENETICS.116.001572 . 27872105 10.1161/CIRCGENETICS.116.001572 PMC5418659

[20] Shrestha S, Irvin MR, Taylor KD, Wiener HW, Pajewski NM, Haritunians T, et al A genome-wide association study of carotid atherosclerosis in HIV-infected men. AIDS. 2010;24(4):583–92. Epub 2009/12/17. doi: 10.1097/QAD.0b013e3283353c9e ; PubMed Central PMCID: PMCPMC3072760. 20009918 10.1097/QAD.0b013e3283353c9e PMC3072760

[21] Shrestha S, Yan Q, Joseph G, Arnett DK, Martinson JJ, Kingsley LA. Replication of RYR3 gene polymorphism association with cIMT among HIV-infected whites. AIDS. 2012;26(12):1571–3. Epub 2012/05/26. doi: 10.1097/QAD.0b013e328355359f ; PubMed Central PMCID: PMCPMC3606588. 22627881 10.1097/QAD.0b013e328355359f PMC3606588

[22] Shendre A, Irvin MR, Aouizerat BE, Wiener HW, Vazquez AI, Anastos K, et al RYR3 gene variants in subclinical atherosclerosis among HIV-infected women in the Women's Interagency HIV Study (WIHS). Atherosclerosis. 2014;233(2):666–72. Epub 2014/02/25. doi: 10.1016/j.atherosclerosis.2014.01.035 ; PubMed Central PMCID: PMCPMC3965606. 24561552 10.1016/j.atherosclerosis.2014.01.035 PMC3965606

[23] Winkler CA, Nelson GW, Smith MW. Admixture mapping comes of age. Annu Rev Genomics Hum Genet. 2010;11:65–89. doi: 10.1146/annurev-genom-082509-141523 . 20594047 10.1146/annurev-genom-082509-141523 PMC7454031

[24] Qin H, Zhu X. Power comparison of admixture mapping and direct association analysis in genome-wide association studies. Genet Epidemiol. 2012;36(3):235–43. doi: 10.1002/gepi.21616 ; PubMed Central PMCID: PMC3589174. 22460597 10.1002/gepi.21616 PMC3589174

[25] Shendre A, Wiener H, Irvin MR, Zhi D, Limdi NA, Overton ET, et al Admixture Mapping of Subclinical Atherosclerosis and Subsequent Clinical Events Among African Americans in 2 Large Cohort Studies. Circ Cardiovasc Genet. 2017;10(2). doi: 10.1161/CIRCGENETICS.116.001569 . 28408707 10.1161/CIRCGENETICS.116.001569 PMC5396391

[26] Shendre A, Irvin MR, Wiener H, Zhi D, Limdi NA, Overton ET, et al Local Ancestry and Clinical Cardiovascular Events Among African Americans From the Atherosclerosis Risk in Communities Study. J Am Heart Assoc. 2017;6(4). doi: 10.1161/JAHA.116.004739 . 28396569 10.1161/JAHA.116.004739 PMC5532995

[27] Price AL, Patterson NJ, Plenge RM, Weinblatt ME, Shadick NA, Reich D. Principal components analysis corrects for stratification in genome-wide association studies. Nat Genet. 2006;38(8):904–9. doi: 10.1038/ng1847 16862161 10.1038/ng1847

[28] Qin H, Morris N, Kang SJ, Li M, Tayo B, Lyon H, et al Interrogating local population structure for fine mapping in genome-wide association studies. Bioinformatics. 2010;26(23):2961–8. doi: 10.1093/bioinformatics/btq560 ; PubMed Central PMCID: PMCPMC2982153. 20889494 10.1093/bioinformatics/btq560 PMC2982153

[29] Lettre G, Palmer CD, Young T, Ejebe KG, Allayee H, Benjamin EJ, et al Genome-wide association study of coronary heart disease and its risk factors in 8,090 African Americans: the NHLBI CARe Project. PLoS Genet. 2011;7(2):e1001300 Epub 2011/02/25. doi: 10.1371/journal.pgen.1001300 ; PubMed Central PMCID: PMCPMC3037413 21347282 10.1371/journal.pgen.1001300 PMC3037413

[30] Pasaniuc B, Zaitlen N, Lettre G, Chen GK, Tandon A, Kao WH, et al Enhanced statistical tests for GWAS in admixed populations: assessment using African Americans from CARe and a Breast Cancer Consortium. PLoS Genet. 2011;7(4):e1001371 doi: 10.1371/journal.pgen.1001371 ; PubMed Central PMCID: PMC3080860. 21541012 10.1371/journal.pgen.1001371 PMC3080860

[31] Shriner D, Adeyemo A, Rotimi CN. Joint ancestry and association testing in admixed individuals. PLoS Comput Biol. 2011;7(12):e1002325 doi: 10.1371/journal.pcbi.1002325 ; PubMed Central PMCID: PMC3245293. 22216000 10.1371/journal.pcbi.1002325 PMC3245293

[32] Zhang J, Stram DO. The role of local ancestry adjustment in association studies using admixed populations. Genet Epidemiol. 2014;38(6):502–15. doi: 10.1002/gepi.21835 . 25043967 10.1002/gepi.21835 PMC5079159

[33] McCormick C, Leduc Y, Martindale D, Mattison K, Esford LE, Dyer AP, et al The putative tumour suppressor EXT1 alters the expression of cell-surface heparan sulfate. Nature genetics. 1998;19(2):158–61. doi: 10.1038/514 . 9620772 10.1038/514

[34] Heinritz W, Huffmeier U, Strenge S, Miterski B, Zweier C, Leinung S, et al New mutations of EXT1 and EXT2 genes in German patients with Multiple Osteochondromas. Annals of human genetics. 2009;73(Pt 3):283–91. doi: 10.1111/j.1469-1809.2009.00508.x . 19344451 10.1111/j.1469-1809.2009.00508.x

[35] Mooij HL, Cabrales P, Bernelot Moens SJ, Xu D, Udayappan SD, Tsai AG, et al Loss of function in heparan sulfate elongation genes EXT1 and EXT 2 results in improved nitric oxide bioavailability and endothelial function. Journal of the American Heart Association. 2014;3(6):e001274 doi: 10.1161/JAHA.114.001274 ; PubMed Central PMCID: PMC4338717. 25468659 10.1161/JAHA.114.001274 PMC4338717

[36] Mittal CK, Jadhav AL. Calcium-dependent inhibition of constitutive nitric oxide synthase. Biochemical and biophysical research communications. 1994;203(1):8–15. doi: 10.1006/bbrc.1994.2141 . 7521166 10.1006/bbrc.1994.2141

[37] Lanner JT, Georgiou DK, Joshi AD, Hamilton SL. Ryanodine receptors: structure, expression, molecular details, and function in calcium release. Cold Spring Harb Perspect Biol. 2010;2(11):a003996 doi: 10.1101/cshperspect.a003996 ; PubMed Central PMCID: PMC2964179. 20961976 10.1101/cshperspect.a003996 PMC2964179

[38] Ruiz A, Pauls E, Badia R, Riveira-Munoz E, Clotet B, Ballana E, et al Characterization of the influence of mediator complex in HIV-1 transcription. J Biol Chem. 2014;289(40):27665–76. doi: 10.1074/jbc.M114.570341 ; PubMed Central PMCID: PMCPMC4183804. 25100719 10.1074/jbc.M114.570341 PMC4183804

[39] Shrestha S, Irvin MR, Grunfeld C, Arnett DK. HIV, Inflammation, and Calcium in Atherosclerosis. Arterioscler Thromb Vasc Biol. 2014;34(2):244–50. doi: 10.1161/ATVBAHA.113.302191 . 24265418 10.1161/ATVBAHA.113.302191

[40] Schiano C, Casamassimi A, Vietri MT, Rienzo M, Napoli C. The roles of mediator complex in cardiovascular diseases. Biochim Biophys Acta. 2014;1839(6):444–51. doi: 10.1016/j.bbagrm.2014.04.012 . 24751643 10.1016/j.bbagrm.2014.04.012

[41] DeBusk LM, Boelte K, Min Y, Lin PC. Heterozygous deficiency of delta-catenin impairs pathological angiogenesis. J Exp Med. 2010;207(1):77–84. doi: 10.1084/jem.20091097 ; PubMed Central PMCID: PMCPMC2812534. 20048286 10.1084/jem.20091097 PMC2812534

[42] Mackinnon MJ, Ndila C, Uyoga S, Macharia A, Snow RW, Band G, et al Environmental Correlation Analysis for Genes Associated with Protection against Malaria. Mol Biol Evol. 2016 doi: 10.1093/molbev/msw004 . 26744416 10.1093/molbev/msw004 PMC4839215

[43] Connelly JJ, Shah SH, Doss JF, Gadson S, Nelson S, Crosslin DR, et al Genetic and functional association of FAM5C with myocardial infarction. BMC Med Genet. 2008;9:33 doi: 10.1186/1471-2350-9-33 ; PubMed Central PMCID: PMCPMC2383879. 18430236 10.1186/1471-2350-9-33 PMC2383879

[44] Sato J, Kinugasa M, Satomi-Kobayashi S, Hatakeyama K, Knox AJ, Asada Y, et al Family with sequence similarity 5, member C (FAM5C) increases leukocyte adhesion molecules in vascular endothelial cells: implication in vascular inflammation. PLoS One. 2014;9(9):e107236 doi: 10.1371/journal.pone.0107236 ; PubMed Central PMCID: PMCPMC4175995. 25251368 10.1371/journal.pone.0107236 PMC4175995

[45] Gegenbauer K, Elia G, Blanco-Fernandez A, Smolenski A. Regulator of G-protein signaling 18 integrates activating and inhibitory signaling in platelets. Blood. 2012;119(16):3799–807. doi: 10.1182/blood-2011-11-390369 . 22234696 10.1182/blood-2011-11-390369

[46] Lang R, Gundlach AL, Kofler B. The galanin peptide family: receptor pharmacology, pleiotropic biological actions, and implications in health and disease. Pharmacology & therapeutics. 2007;115(2):177–207. Epub 2007/07/03. doi: 10.1016/j.pharmthera.2007.05.009 . 17604107 10.1016/j.pharmthera.2007.05.009

[47] Touyz RM. Transient receptor potential melastatin 6 and 7 channels, magnesium transport, and vascular biology: implications in hypertension. Am J Physiol Heart Circ Physiol. 2008;294(3):H1103–18. doi: 10.1152/ajpheart.00903.2007 . 18192217 10.1152/ajpheart.00903.2007

[48] Fox ER, Musani SK, Barbalic M, Lin H, Yu B, Ogunyankin KO, et al Genome-wide association study of cardiac structure and systolic function in African Americans: the Candidate Gene Association Resource (CARe) study. Circ Cardiovasc Genet. 2013;6(1):37–46. doi: 10.1161/CIRCGENETICS.111.962365 ; PubMed Central PMCID: PMCPMC3591479. 23275298 10.1161/CIRCGENETICS.111.962365 PMC3591479

[49] GeneCards. Pathways & Interactions for PLPP4 Gene [cited 2016 03/23/2016]. Available from: http://www.genecards.org/cgi-bin/carddisp.pl?gene=PLPP4-pathways_interactions.

[50] Kaplan RC, Kingsley LA, Gange SJ, Benning L, Jacobson LP, Lazar J, et al Low CD4+ T-cell count as a major atherosclerosis risk factor in HIV-infected women and men. AIDS. 2008;22(13):1615–24. Epub 2008/08/02. doi: 10.1097/QAD.0b013e328300581d ; PubMed Central PMCID: PMCPmc2624572. 18670221 10.1097/QAD.0b013e328300581d PMC2624572

[51] Seaberg EC, Benning L, Sharrett AR, Lazar JM, Hodis HN, Mack WJ, et al Association between human immunodeficiency virus infection and stiffness of the common carotid artery. Stroke. 2010;41(10):2163–70. doi: 10.1161/STROKEAHA.110.583856 ; PubMed Central PMCID: PMCPMC2972735. 20798374 10.1161/STROKEAHA.110.583856 PMC2972735

[52] Devlin B, Roeder K. Genomic control for association studies. Biometrics. 1999;55(4):997–1004. 11315092 10.1111/j.0006-341x.1999.00997.x

[53] Yorgov D, Edwards KL, Santorico SA. Use of admixture and association for detection of quantitative trait loci in the Type 2 Diabetes Genetic Exploration by Next-Generation Sequencing in Ethnic Samples (T2D-GENES) study. BMC Proc. 2014;8(Suppl 1):S6 doi: 10.1186/1753-6561-8-S1-S6 ; PubMed Central PMCID: PMCPMC4143673. 25519335 10.1186/1753-6561-8-S1-S6 PMC4143673

[54] Barkan SE, Melnick SL, Preston-Martin S, Weber K, Kalish LA, Miotti P, et al The Women's Interagency HIV Study. WIHS Collaborative Study Group. Epidemiology. 1998;9(2):117–25. Epub 1998/03/21. . 9504278

[55] Bacon MC, von Wyl V, Alden C, Sharp G, Robison E, Hessol N, et al The Women's Interagency HIV Study: an observational cohort brings clinical sciences to the bench. Clin Diagn Lab Immunol. 2005;12(9):1013–9. doi: 10.1128/CDLI.12.9.1013-1019.2005 ; PubMed Central PMCID: PMC1235804. 16148165 10.1128/CDLI.12.9.1013-1019.2005 PMC1235804

[56] Hodis HN, Mack WJ, Lobo RA, Shoupe D, Sevanian A, Mahrer PR, et al Estrogen in the prevention of atherosclerosis. A randomized, double-blind, placebo-controlled trial. Ann Intern Med. 2001;135(11):939–53. . 11730394 10.7326/0003-4819-135-11-200112040-00005

[57] Selzer RH, Mack WJ, Lee PL, Kwong-Fu H, Hodis HN. Improved common carotid elasticity and intima-media thickness measurements from computer analysis of sequential ultrasound frames. Atherosclerosis. 2001;154(1):185–93. http://dx.doi.org/10.1016/S0021-9150(00)00461-5. 11137099 10.1016/s0021-9150(00)00461-5

[58] Feldman DN, Feldman JG, Greenblatt R, Anastos K, Pearce L, Cohen M, et al CYP1A1 genotype modifies the impact of smoking on effectiveness of HAART among women. AIDS Educ Prev. 2009;21(3 Suppl):81–93. doi: 10.1521/aeap.2009.21.3_supp.81 ; PubMed Central PMCID: PMC2754267. 19537956 10.1521/aeap.2009.21.3_supp.81 PMC2754267

[59] Purcell S, Neale B, Todd-Brown K, Thomas L, Ferreira MAR, Bender D, et al PLINK: a tool set for whole-genome association and population-based linkage analyses. Am J Hum Genet. 2007;81:559–75. doi: 10.1086/519795 . 17701901 10.1086/519795 PMC1950838

[60] Frasco MA, Mack WJ, Van Den Berg D, Aouizerat BE, Anastos K, Cohen M, et al Underlying genetic structure impacts the association between CYP2B6 polymorphisms and response to efavirenz and nevirapine. AIDS. 2012;26(16):2097–106. doi: 10.1097/QAD.0b013e3283593602 . 22951632 10.1097/QAD.0b013e3283593602 PMC3940150

[61] Smith MW, Patterson N, Lautenberger JA, Truelove AL, McDonald GJ, Waliszewska A, et al A High-Density Admixture Map for Disease Gene Discovery in African Americans. Am J Hum Genet. 2004;74(5):1001–13. doi: 10.1086/420856 15088270 10.1086/420856 PMC1181963

[62] Baran Y, Pasaniuc B, Sankararaman S, Torgerson DG, Gignoux C, Eng C, et al Fast and accurate inference of local ancestry in Latino populations. Bioinformatics. 2012;28(10):1359–67. doi: 10.1093/bioinformatics/bts144 ; PubMed Central PMCID: PMC3348558. 22495753 10.1093/bioinformatics/bts144 PMC3348558

[63] Karolchik D, Hinrichs AS, Furey TS, Roskin KM, Sugnet CW, Haussler D, et al The UCSC Table Browser data retrieval tool. Nucleic Acids Res. 2004;32(Database issue):D493–6. doi: 10.1093/nar/gkh103 ; PubMed Central PMCID: PMCPMC308837. 14681465 10.1093/nar/gkh103 PMC308837

[64] Chang X, Wang K. wANNOVAR: annotating genetic variants for personal genomes via the web. J Med Genet. 2012;49(7):433–6. doi: 10.1136/jmedgenet-2012-100918 ; PubMed Central PMCID: PMCPMC3556337. 22717648 10.1136/jmedgenet-2012-100918 PMC3556337

[65] Gomez F, Wang L, Abel H, Zhang Q, Province MA, Borecki IB. Admixture mapping of coronary artery calcification in African Americans from the NHLBI family heart study. BMC Genet. 2015;16:42 doi: 10.1186/s12863-015-0196-x ; PubMed Central PMCID: PMCPMC4417236. 25902833 10.1186/s12863-015-0196-x PMC4417236

[66] Hessler N, Geisel MH, Coassin S, Erbel R, Heilmann S, Hennig F, et al Linkage and Association Analysis Identifies TRAF1 Influencing Common Carotid Intima-Media Thickness. Stroke. 2016;47(12):2904–9. Epub 2016/11/09. doi: 10.1161/STROKEAHA.116.013943 . 27827325 10.1161/STROKEAHA.116.013943

[67] Chen YC, Hsu KH, Chiou HY, Juang YL, Wu TW, Hung CL, et al Associations of lipid-related genetic markers with elevated carotid intima-media thickness in middle-age adults and elders. Int J Cardiol. 2015;189:264–6. doi: 10.1016/j.ijcard.2015.04.034 . 25902421 10.1016/j.ijcard.2015.04.034

[68] Iemitsu M, Fujie S, Murakami H, Sanada K, Kawano H, Gando Y, et al Higher cardiorespiratory fitness attenuates the risk of atherosclerosis associated with ADRB3 Trp64Arg polymorphism. Eur J Appl Physiol. 2014;114(7):1421–8. Epub 2014/03/25. doi: 10.1007/s00421-014-2862-5 . 24658877 10.1007/s00421-014-2862-5

[69] Bis JC, White CC, Franceschini N, Brody J, Zhang X, Muzny D, et al Sequencing of 2 subclinical atherosclerosis candidate regions in 3669 individuals: Cohorts for Heart and Aging Research in Genomic Epidemiology (CHARGE) Consortium Targeted Sequencing Study. Circ Cardiovasc Genet. 2014;7(3):359–64. Epub 2014/06/22. doi: 10.1161/CIRCGENETICS.113.000116 ; PubMed Central PMCID: PMCPMC4112104. 24951662 10.1161/CIRCGENETICS.113.000116 PMC4112104

[70] Zhao J, Goldberg J, Vaccarino V. Leukotriene A4 hydrolase haplotype, diet and atherosclerosis: a twin study. Atherosclerosis. 2013;226(1):238–44. Epub 2012/11/17. doi: 10.1016/j.atherosclerosis.2012.10.048 ; PubMed Central PMCID: PMCPMC3630507. 23153620 10.1016/j.atherosclerosis.2012.10.048 PMC3630507

[71] Markus HS, Makela KM, Bevan S, Raitoharju E, Oksala N, Bis JC, et al Evidence HDAC9 genetic variant associated with ischemic stroke increases risk via promoting carotid atherosclerosis. Stroke. 2013;44(5):1220–5. Epub 2013/03/02. doi: 10.1161/STROKEAHA.111.000217 ; PubMed Central PMCID: PMCPMC4206941. 23449258 10.1161/STROKEAHA.111.000217 PMC4206941

[72] Wang L, Beecham A, Zhuo D, Dong C, Blanton SH, Rundek T, et al Fine Mapping Study Reveals Novel Candidate Genes for Carotid Intima-Media Thickness in Dominican Families. Circ Cardiovasc Genet. 2012 Epub 2012/03/17. doi: 10.1161/circgenetics.111.961763 . 22423143 10.1161/CIRCGENETICS.111.961763 PMC3341091

[73] Wassel CL, Pankow JS, Rasmussen-Torvik LJ, Li N, Taylor KD, Guo X, et al Associations of SNPs in ADIPOQ and subclinical cardiovascular disease in the multi-ethnic study of atherosclerosis (MESA). Obesity. 2011;19(4):840–7. Epub 2010/10/12. doi: 10.1038/oby.2010.229 . 20930713 10.1038/oby.2010.229 PMC3510267

[74] Rahman TJ, Walker EA, Mayosi BM, Hall DH, Avery PJ, Connell JM, et al Genotype at the P554L variant of the hexose-6 phosphate dehydrogenase gene is associated with carotid intima-medial thickness. PLoS One. 2011;6(8):e23248 doi: 10.1371/journal.pone.0023248 ; PubMed Central PMCID: PMCPMC3155541. 21858044 10.1371/journal.pone.0023248 PMC3155541

[75] Goodarzi MO, Taylor KD, Jones MR, Fang B, Guo X, Xiang AH, et al Replication of calpain-10 genetic association with carotid intima-media thickness. Atherosclerosis. 2009;205(2):503–5. Epub 2009/02/06. doi: 10.1016/j.atherosclerosis.2008.12.038 ; PubMed Central PMCID: PMCPMC2717175. 19193380 10.1016/j.atherosclerosis.2008.12.038 PMC2717175

[76] Debette S, Bevan S, Dartigues JF, Sitzer M, Lorenz M, Ducimetiere P, et al Fractalkine receptor/ligand genetic variants and carotid intima-media thickness. Stroke. 2009;40(6):2212–4. Epub 2009/04/18. doi: 10.1161/STROKEAHA.108.537159 . 19372452 10.1161/STROKEAHA.108.537159

[77] Liao YC, Lin HF, Rundek T, Cheng R, Guo YC, Sacco RL, et al Segment-specific genetic effects on carotid intima-media thickness: the Northern Manhattan study. Stroke. 2008;39(12):3159–65. Epub 2008/09/13. doi: 10.1161/STROKEAHA.108.522789 ; PubMed Central PMCID: PMC2676928. 18787196 10.1161/STROKEAHA.108.522789 PMC2676928

[78] O'Donnell CJ, Cupples LA, D'Agostino RB, Fox CS, Hoffmann U, Hwang SJ, et al Genome-wide association study for subclinical atherosclerosis in major arterial territories in the NHLBI's Framingham Heart Study. BMC Med Genet. 2007;8 Suppl 1:S4 Epub 2007/10/16. doi: 10.1186/1471-2350-8-s1-s4 ; PubMed Central PMCID: PMCPmc1995605. 17903303 10.1186/1471-2350-8-S1-S4 PMC1995605

[79] Dumont J, Zureik M, Cottel D, Montaye M, Ducimetiere P, Amouyel P, et al Association of arginase 1 gene polymorphisms with the risk of myocardial infarction and common carotid intima media thickness. J Med Genet. 2007;44(8):526–31. Epub 2007/03/21. doi: 10.1136/jmg.2006.047449 ; PubMed Central PMCID: PMCPMC2597928. 17369504 10.1136/jmg.2006.047449 PMC2597928

[80] Dumont J, Zureik M, Bauters C, Grupposo MC, Cottel D, Montaye M, et al Association of OAZ1 gene polymorphisms with subclinical and clinical vascular events. Arterioscler Thromb Vasc Biol. 2007;27(10):2120–6. Epub 2007/09/01. doi: 10.1161/ATVBAHA.107.150458 . 17761941 10.1161/ATVBAHA.107.150458

[81] Bhuiyan AR, Chen W, Srinivasan SR, Rice J, Mock N, Tang R, et al Influence of nitric oxide synthase gene polymorphism (G894T) on carotid artery intima-media thickness in adults: the Bogalusa Heart Study. J Am Soc Hypertens. 2007;1(5):362–8. Epub 2007/09/01. doi: 10.1016/j.jash.2007.06.006 . 20409867 10.1016/j.jash.2007.06.006

[82] Yazdanpanah M, Sayed-Tabatabaei FA, Hofman A, Aulchenko YS, Oostra BA, Stricker BH, et al The alpha-adducin gene is associated with macrovascular complications and mortality in patients with type 2 diabetes. Diabetes. 2006;55(10):2922–7. Epub 2006/09/28. doi: 10.2337/db06-0302 . 17003363 10.2337/db06-0302

[83] Pollex RL, Hegele R. Genetic determinants of carotid ultrasound traits. Curr Atheroscler Rep. 2006;8(3):206–15. doi: 10.1007/s11883-006-0075-z . 16640957 10.1007/s11883-006-0075-z

[84] Coll B, Parra S, Alonso-Villaverde C, de Groot E, Aragones G, Montero M, et al HIV-infected patients with lipodystrophy have higher rates of carotid atherosclerosis: the role of monocyte chemoattractant protein-1. Cytokine. 2006;34(1–2):51–5. Epub 2006/05/16. doi: 10.1016/j.cyto.2006.03.013 . 16697654 10.1016/j.cyto.2006.03.013

[85] Coll B, Alonso-Villaverde C, Parra S, Montero M, Tous M, Joven J, et al The stromal derived factor-1 mutated allele (SDF1-3′A) is associated with a lower incidence of atherosclerosis in HIV-infected patients. AIDS. 2005;19(16):1877–83. 16227796 10.1097/01.aids.0000183516.22266.dd

[86] Manolio TA, Boerwinkle E, O'Donnell CJ, Wilson AF. Genetics of ultrasonographic carotid atherosclerosis. Arterioscler Thromb Vasc Biol. 2004;24(9):1567–77. doi: 10.1161/01.ATV.0000138789.11433.c1 . 15256397 10.1161/01.ATV.0000138789.11433.c1

[87] Kiechl S, Lorenz E, Reindl M, Wiedermann CJ, Oberhollenzer F, Bonora E, et al Toll-like receptor 4 polymorphisms and atherogenesis. N Engl J Med. 2002;347(3):185–92. doi: 10.1056/NEJMoa012673 . 12124407 10.1056/NEJMoa012673

