# Supplementary material for: Genome-wide admixture and association study of subclinical atherosclerosis in the Women’s Interagency HIV Study (WIHS)
Source: PLoS One. 2017 Dec 4;12(12):e0188725. doi: 10.1371/journal.pone.0188725 (PMC5714351; doi:10.1371/journal.pone.0188725)
Supplement: S1 File — Comparing test performance after ancestry adjustment. (DOCX) [file pone.0188725.s001.docx]

**Genome-wide admixture and association study of subclinical atherosclerosis in the Women’s Interagency HIV Study (WIHS)**

**Supplemental methods**

Aditi Shendre,^1^ Howard W. Wiener,^1^ Marguerite R. Irvin,^1^ Bradley E. Aouizerat,^2,3^ Edgar T. Overton,^4^ Kathryn Anastos,^5,14^ Jason Lazar,^6^ Chenglong Liu,^7^ Roksana Karim,^8^ Nita A. Limdi,^9^ Mardge H. Cohen,^10^ Elizabeth T. Golub,^11^ Degui Zhi,^12^ Daniel Shriner,^13^ Qibin Qi,^14^ David B. Hanna,^14^ Robert C. Kaplan,^14^ Sadeep Shrestha,*^1^

^1^Department of Epidemiology, University of Alabama at Birmingham, Birmingham, AL, USA

^2^Bluestone Center for Clinical Research, New York University, NY, USA

^3^Department of Oral and Maxillofacial Surgery, New York University, NY, USA

^4^Division of Infectious Diseases, Department of Medicine, University of Alabama at Birmingham, Birmingham, AL, USA;

^5^Department of Medicine, Albert Einstein College of Medicine, Bronx, NY, USA

^6^Department of Medicine, State University of New York, Downstate Medical Center, Brooklyn, NY, USA

^7^Department of Medicine, Georgetown University Medical Center, Washington, DC, USA

^8^Atherosclerosis Research Unit, University of Southern California, Los Angeles, CA, USA

^9^Department of Neurology, University of Alabama at Birmingham, Birmingham, AL, USA

^10^Department of Medicine, John Stroger Hospital and Rush University, Chicago, IL, USA

^11^Department of Epidemiology, Johns Hopkins Bloomberg School of Public Health, Baltimore, MD, USA

^12^Department of Biostatistics, University of Alabama at Birmingham, Birmingham, AL, USA

^13^Center for Research on Genomics and Global Health, National Human Genome Research Institute, Bethesda, MD, USA

^14^Department of Epidemiology and Population Health, Albert Einstein College of Medicine, Bronx, NY, USA

**Correspondence:**

^*^Sadeep Shrestha, PhD MHS MS

University of Alabama at Birmingham

School of Public Health

Department of Epidemiology, Ryals Rm217L

1720 2nd Ave S., Birmingham, AL 35294-0022

Ph: 205-934-6459; email: [sshrestha@uab.edu](mailto:sshrestha@uab.edu)

**Comparing test performance after ancestry adjustment**

In the subset of SNPs that were examined for local ancestry association (n=473,732), we also tested whether the single-SNP associations improved after adjusting for ancestry information. We employed the MIXSCORE (v1.3) program developed by Pasaniuc et al. [1] for quantitative traits to determine statistical power for each test. The tests include the 1 degree of freedom (df) Armitage trend test (QATT) for genotype data adjusted for global ancestry, a 1 df SNP association test adjusted for local ancestry (QSNP1), a 1 df test to determine admixture association at each SNP (QADM), and a 2 df test (QSUM) which is the sum of the chi-square statistics from QSNP1 and QADM, respectively. The results for all the above-mentioned tests were based on bivariate analyses. We also performed joint admixture and association testing using a Bayesian approach (BMIX) as proposed by Shriner et al [2]. The BMIX method estimates the joint posterior probability for each SNP by initially estimating the local ancestry conditional on global ancestry, followed by association analysis stratified on local ancestry. The joint probability is calculated from the pooled estimates of the local ancestry strata using the posterior probability of admixture as the prior for association analysis. The statistical power for BMIX was estimated using the posterior probability from the overall and stratified analyses, respectively.

**References**

1. Pasaniuc B, Zaitlen N, Lettre G, Chen GK, Tandon A, et al. (2011) Enhanced statistical tests for GWAS in admixed populations: assessment using African Americans from CARe and a Breast Cancer Consortium. PLoS Genet 7: e1001371.

2. Shriner D, Adeyemo A, Rotimi CN (2011) Joint ancestry and association testing in admixed individuals. PLoS Comput Biol 7: e1002325.
