# Supplementary material for: Genome-wide admixture and association study of subclinical atherosclerosis in the Women’s Interagency HIV Study (WIHS)
Source: PLoS One. 2017 Dec 4;12(12):e0188725. doi: 10.1371/journal.pone.0188725 (PMC5714351; doi:10.1371/journal.pone.0188725)
Supplement: S3 Table — QATT: quantitative Armitage’s trend test adjusting for global ancestry, QSNP1: quantitative single nucleotide polymorphism association adjusting for local ancestry, QADM: quantitative admixture test, QSUM: sum of QSNP1 and QADM, BMIX: joint admixture and association test using a Bayesian approach, Score: minus log 10 p-values. (DOCX) [file pone.0188725.s004.docx]

**S3 Table. Comparing test performance after adjusting for global, local or both ancestries in the association tests.**

|  |  | **QATT (1df)** | **QSNP1 (1df)** | **QADM (1df)** | **QSUM (2df)** | **BMIX (1df)** |
| --- | --- | --- | --- | --- | --- | --- |
| All | Chi-square | 2.7623 | 2.7544 | 2.9797 | 9.6135 | 17.4841 |
|  | P Value | 0.0965 | 0.0970 | 0.0843 | 0.0082 | 0.00003 |
|  | Score | 1.0154 | 1.0133 | 1.0741 | 2.0875 | 4.5229 |
|  | Power | 0.0002 | 0.0002 | 0.0318 | 0.2263 | 0.7258 |
| HIV positive | Chi-square | 2.7559 | 2.7479 | 3.0951 | 9.7493 | 16.2005 |
|  | P Value | 0.0969 | 0.0974 | 0.0785 | 0.0076 | 0.00006 |
|  | Score | 1.0137 | 1.0115 | 1.1050 | 2.1170 | 4.2218 |
|  | Power | 0.0002 | 0.0002 | 0.0342 | 0.2328 | 0.6714 |
| HIV negative | Chi-square | 2.4790 | 2.7509 | 2.9952 | 9.6266 | 8.3298 |
|  | P Value | 0.0973 | 0.0972 | 0.0835 | 0.0081 | 0.0039 |
|  | Score | 1.0118 | 1.0123 | 1.0782 | 2.0904 | 2.4089 |
|  | Power | 0.0001 | 0.0002 | 0.0321 | 0.2269 | 0.2435 |

QATT: quantitative Armitage’s trend test adjusting for global ancestry, QSNP1: quantitative single nucleotide polymorphism association adjusting for local ancestry, QADM: quantitative admixture test, QSUM: sum of QSNP1 and QADM, BMIX: joint admixture and association test using a Bayesian approach, Score: minus log 10 p-values
