# Supplementary material for: Genome-wide admixture and association study of subclinical atherosclerosis in the Women’s Interagency HIV Study (WIHS)
Source: PLoS One. 2017 Dec 4;12(12):e0188725. doi: 10.1371/journal.pone.0188725 (PMC5714351; doi:10.1371/journal.pone.0188725)
Supplement: S5 Table — SNP: single nucleotide polymorphism, MAF: minor allele frequency. (DOCX) [file pone.0188725.s006.docx]

**S5 Table. Quality control of the genome wide data from the Women’s Interagency HIV study (WIHS)**

| **Chromosome** | **SNPs** | **After hg19 to hg18** | **Not in HAPMAP** | **AT/GC w MAF> 0.35 removed** | **Total removed** | **Duplicates** | **Final after removing duplicates** |
| --- | --- | --- | --- | --- | --- | --- | --- |
| 1 | 102823 | 102823 | 56650 | 104 | 56754 | 25 | 46044 |
| 2 | 74908 | 74898 | 43982 | 74 | 44056 | 0 | 30842 |
| 3 | 63506 | 63499 | 37672 | 111 | 37783 | 0 | 25716 |
| 4 | 86047 | 86032 | 52869 | 68 | 52937 | 9 | 33086 |
| 5 | 81711 | 81711 | 47335 | 80 | 47415 | 0 | 34289 |
| 6 | 54790 | 54788 | 30888 | 64 | 30952 | 1 | 23835 |
| 7 | 73130 | 73100 | 42813 | 62 | 43153 | 9 | 29945 |
| 8 | 49488 | 49460 | 28973 | 72 | 29045 | 0 | 20415 |
| 9 | 59653 | 59653 | 33603 | 88 | 33691 | 5 | 25957 |
| 10 | 47211 | 47211 | 26839 | 70 | 26909 | 0 | 20302 |
| 11 | 65032 | 65032 | 36916 | 59 | 36975 | 3 | 28054 |
| 12 | 63164 | 63156 | 35804 | 57 | 35861 | 5 | 27290 |
| 13 | 47523 | 47522 | 27230 | 48 | 27278 | 1 | 20243 |
| 14 | 30695 | 30694 | 17648 | 41 | 17689 | 0 | 13005 |
| 15 | 42529 | 42529 | 24481 | 53 | 24534 | 0 | 17995 |
| 16 | 32606 | 32606 | 19320 | 71 | 19391 | 0 | 13215 |
| 17 | 38352 | 38335 | 21912 | 40 | 21952 | 2 | 16381 |
| 18 | 29214 | 29214 | 17045 | 25 | 17070 | 0 | 12144 |
| 19 | 20268 | 20268 | 12076 | 24 | 12100 | 1 | 8167 |
| 20 | 33295 | 33295 | 18332 | 38 | 18370 | 2 | 14923 |
| 21 | 13869 | 13869 | 8098 | 25 | 8123 | 0 | 5746 |
| 22 | 14487 | 14487 | 8328 | 21 | 8349 | 0 | 6138 |
| Total | 1124301 | 1124182 | 648814 | 1295 | 650387 | 63 | 473732 |

SNP: single nucleotide polymorphism, MAF: minor allele frequency
