# Supplementary figures and images for: Genome-wide admixture and association study of subclinical atherosclerosis in the Women’s Interagency HIV Study (WIHS)
Source: PLoS One. 2017 Dec 4;12(12):e0188725. doi: 10.1371/journal.pone.0188725 (PMC5714351; doi:10.1371/journal.pone.0188725)

Plotted SNPs

a)

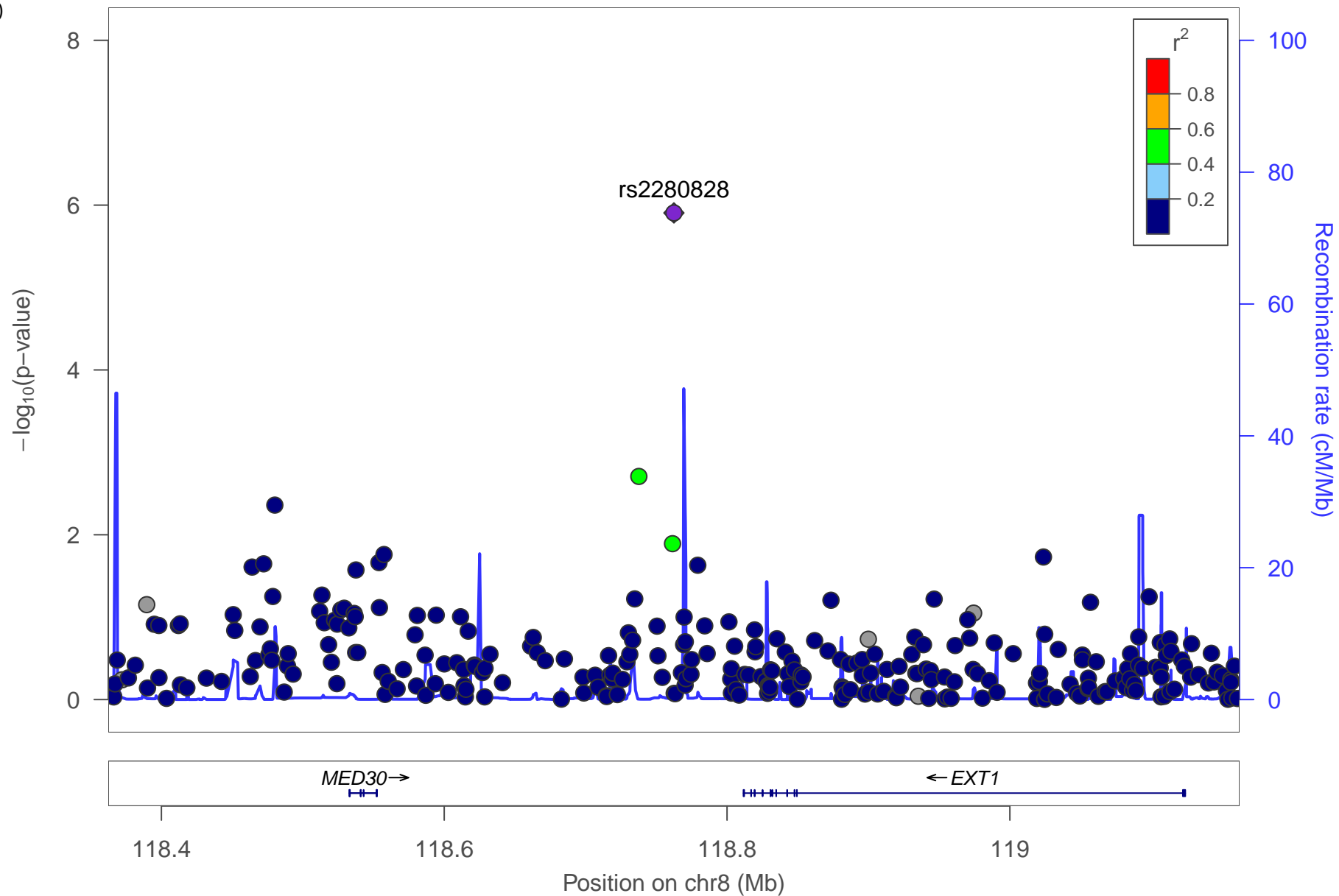

Plotted SNPs

b)

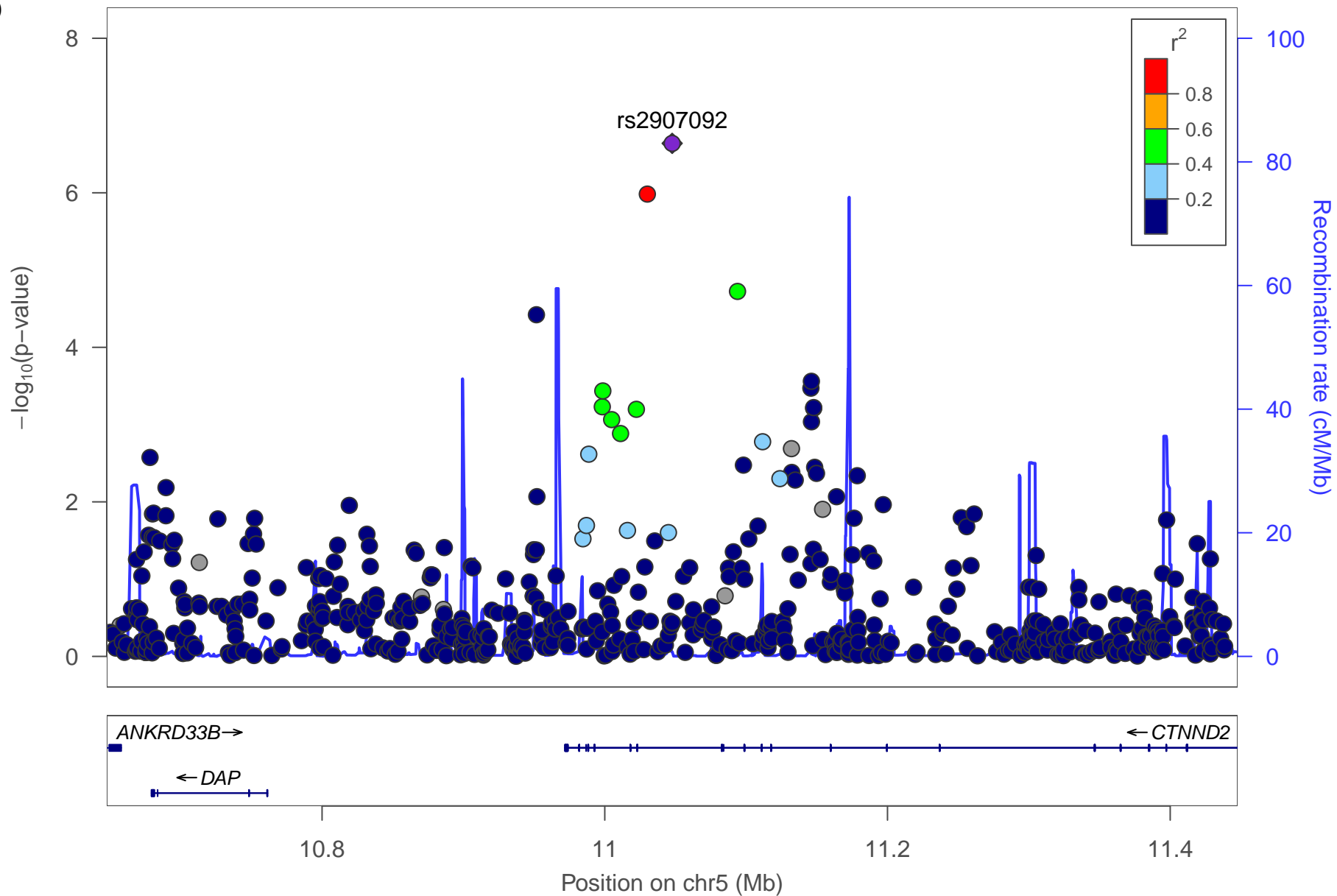

Plotted SNPs

c)

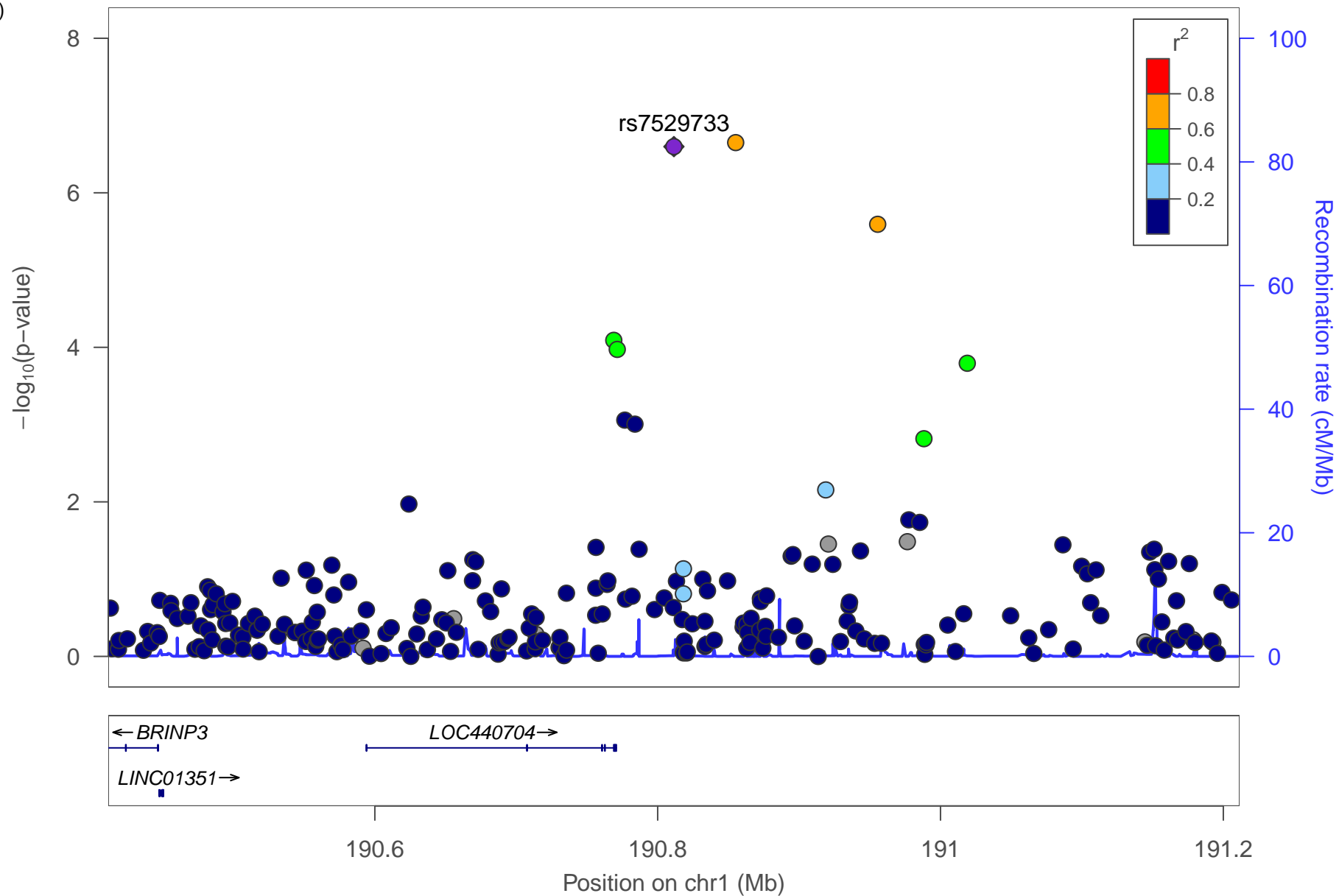

Plotted SNPs | | | | | | | | | | | | | | | | | |

d)

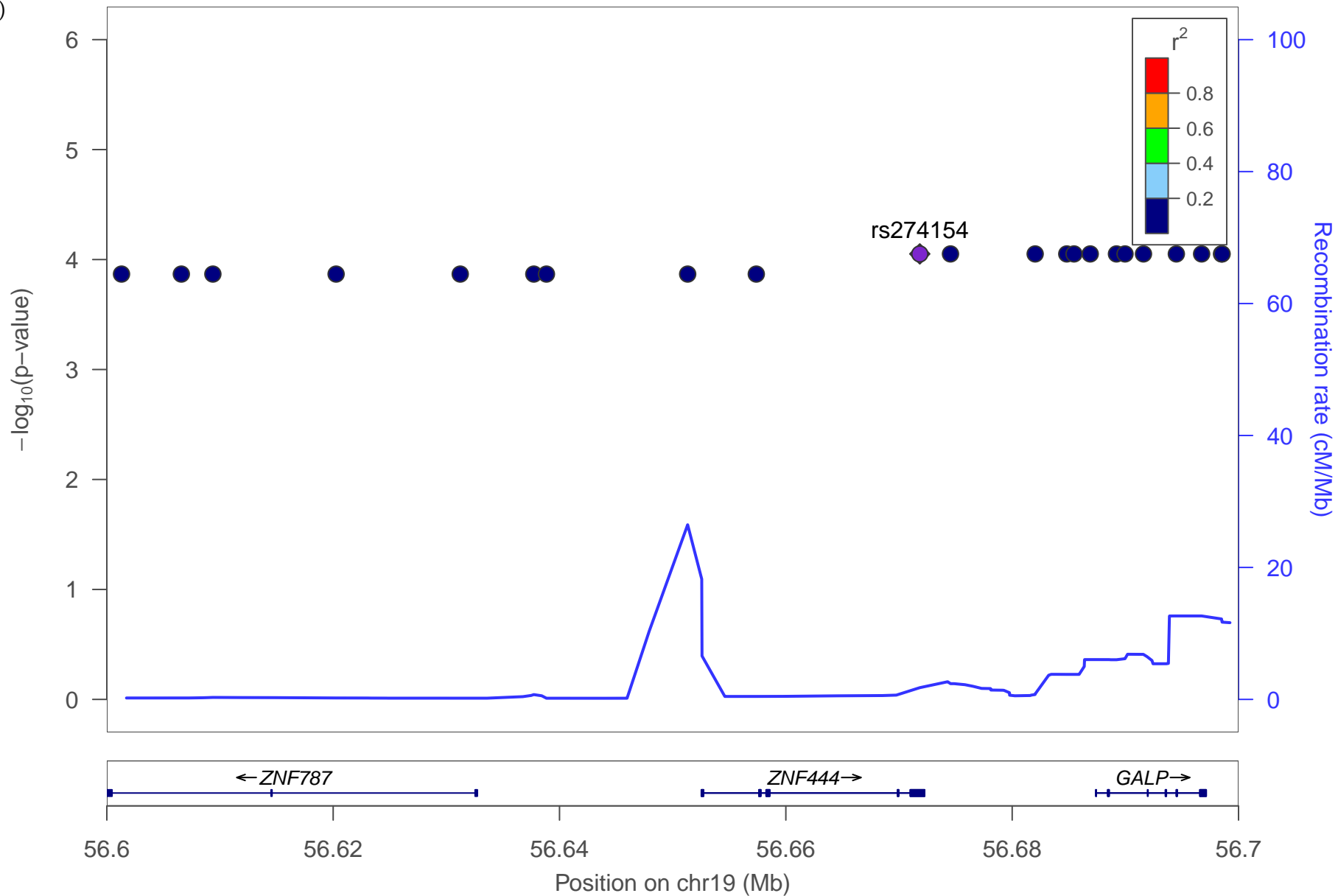

Plotted SNPs

e)

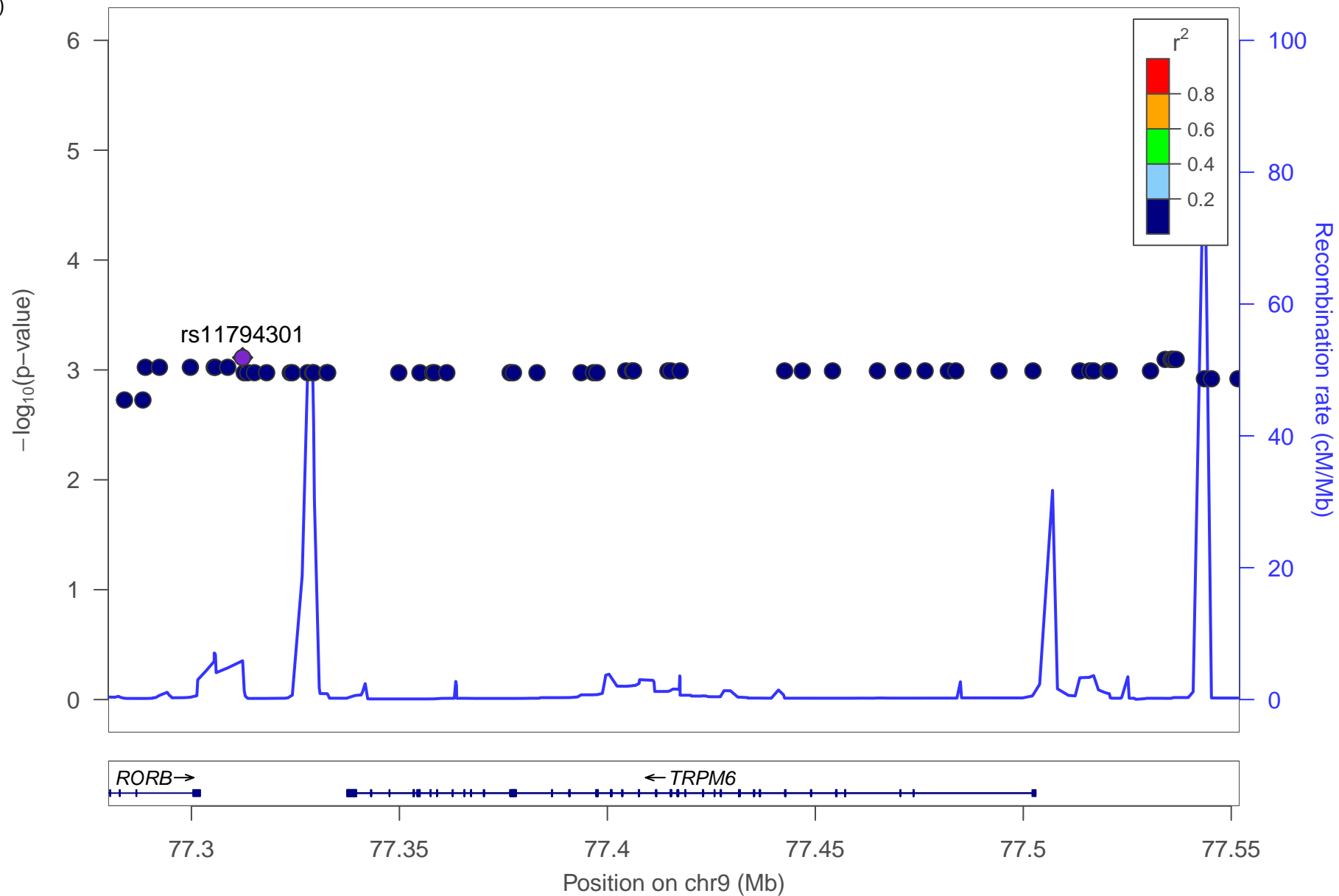

Plotted SNPs

f)

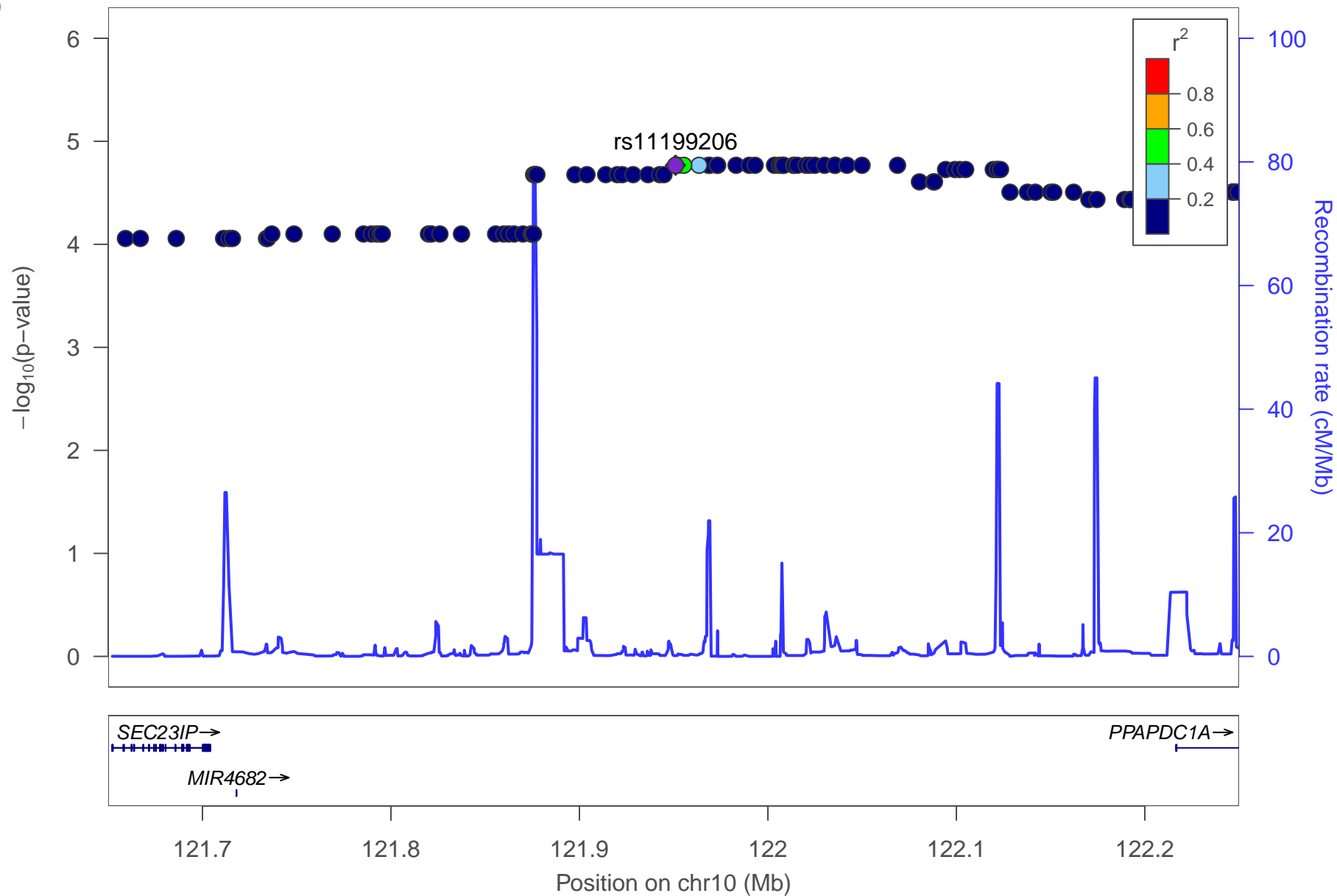

Supplement: S1 Fig — a) All women (GWAS), b) HIV-positive women (GWAS), c) HIV-negative women (GWAS), d) All women (admixture), e) HIV-positive women (admixture), and f) HIV-negative women (admixture). Negative log10 p-values and recombination rates are plotted against SNPs and their chromosomal positions. (PDF) [file pone.0188725.s008.pdf]

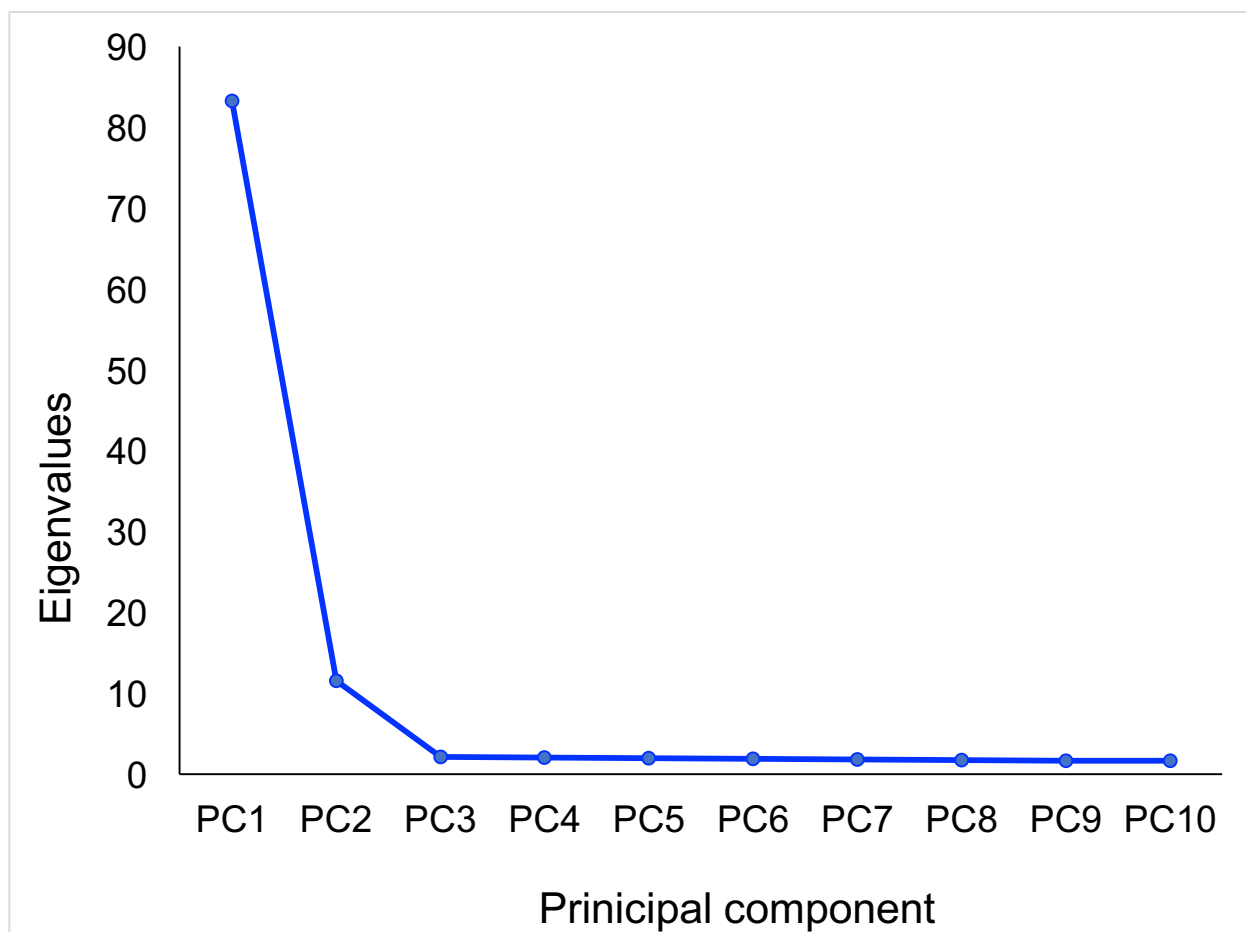

Supplement: S2 Fig — (PDF) [file pone.0188725.s009.pdf]
